# Effect of the Proteasome Inhibitor, Bortezomib, on Histone Modifications in Human Leukemic Cell Lines

**DOI:** 10.3390/ijms27156597

**Published:** 2026-07-24

**Authors:** Hedieh Sattarifard, Marvellous Oyeyode, Dhanvi Prajapati, Angela Duaqui, Gurlovleen Kaur, Ishdeep Muker, Wenxia Luo, Ted M. Lakowski, James R. Davie

**Affiliations:** 1 Department of Biochemistry and Medical Genetics, Rady Faculty of Health Sciences, Max Rady College of Medicine, University of Manitoba, Winnipeg, MB R3E 0J9, Canada; sattarih@myumanitoba.ca (H.S.); oyeyodem@myumanitoba.ca (M.O.); duaquia@myumanitoba.ca (A.D.); kaurg46@myumanitoba.ca (G.K.); mukeri@myumanitoba.ca (I.M.); 2 Pharmaceutical Analysis Laboratory, College of Pharmacy, University of Manitoba, Winnipeg, MB R3E 0T5, Canada; luow1@myumanitoba.ca (W.L.); ted.lakowski@umanitoba.ca (T.M.L.)

**Keywords:** proteasome, bortezomib, histone H2B ubiquitinated at lysine 120, histone H2A ubiquitinated at lysine 119

## Abstract

Histone-modifying enzymes and histone post-translational modifications (PTMs) play key roles in the organization (euchromatin versus heterochromatin) and function (active versus silenced genes) of chromatin. The abundance and activity of these enzymes, along with their associated histone PTMs, are often altered in cancer cells, leading to deregulated gene expression. The expression of the KMT2A-MLLT3 protein, resulting from a chromosomal translocation in mixed-lineage leukemia (MLL), a subtype of acute myeloid leukemia, augments transcription elongation, promoting the expression of *HOXA9* and *MEIS1*, genes that play critical roles in MLL development. Bortezomib, a proteasome inhibitor, has been effective at treating various cancers. In this study, we compared the impact of bortezomib on histone PTMs in the MLL cell line MOLM-13 and the chronic myeloid leukemic (CML) cell line K562. We report that MOLM-13 had a greater level of histone H2B monoubiquitinated at lysine 120 (H2BK120ub) and histone H3 dimethylated at lysine 79 (H3K79me2) (modifications involved in elongation) and similar levels of histone H2A monoubiquitinated at lysine 119 (H2AK119ub). Bortezomib treatment resulted in significant reductions in H2BK120ub and H2AK119ub levels, as well as in transcript levels of genes involved in MLL development.

## 1. Introduction

Acute myeloid leukemia (AML) is an aggressive blood cancer that affects both adults and children but mainly occurs in pediatric patients. MLL, a subtype of AML, is characterized by a *KMT2A* rearrangement at 11q23 that can fuse with over 80 partner genes, including but not limited to AFF1/AF4, MLLT10/AF10, MLLT3/AF9, and MLLT1/EN [1]. The KMT2A-MLLT3 fusion protein, resulting from the t(9;11)(p22;q23) translocation, is one of the most common and well-studied fusions in *KMT2A*-rearranged leukemia. In AML, the *KMT2A* gene, which codes for an H3K4 lysine methyltransferase, fuses with another protein-coding gene, generating an oncoprotein. In this fusion, *KMT2A* retains its *N*-terminus (KMT2A^N^), which is placed in frame with the *C*-terminus of one of several fusion partners, creating an oncogenic chimeric protein. In this translocation, although KMT2A does not contain the H3K4 methylation SET domain, it does contain a CXXC domain that can bind to nonmethylated CpG-rich promoter regions of target genes of KMT2A, like the *HOXA9* gene. Upon binding to CpG regions, the fusion protein can recruit DOT1L (KMT4), the Super Elongation Complex, and other chromatin-modifying enzymes involved in transcription elongation, thereby increasing the rate of elongation of target genes such as *HOXA9* [2]. The Polycomb Repressive Complex 1.1, which deposits H2AK119ub at promoter regions of transcribed genes, also plays a critical role in KMT2A-rearranged leukemias [3,4].

The 26S proteasome is a large proteolytic complex responsible for the degradation and recycling of proteins, a process that requires metabolic energy. It recognizes, unfolds, and cleaves proteins destined for removal, usually by prior attachment to ubiquitin polymers, such as the 76-amino-acid protein with a molecular mass of approximately 8.5 kDa. This tagging process, called ubiquitination, involves a cascade of enzymes: E1 (ubiquitin-activating enzyme), E2 (ubiquitin-conjugating enzyme), and E3 (ubiquitin-ligase enzyme) [5]. The 26S proteasome macromolecular machine comprises two subcomplexes, the 19S regulatory particle and the 20S core particle. The 20S proteasome is the catalytic part of the 26S proteasome involved in the proteolysis of proteins marked for degradation by the ubiquitin system [5].

Proteasomes are classified into two main groups: the constitutive proteasome and the immunoproteasome. The constitutive proteasome is present in almost all cell types and degrades intracellular proteins under normal physiological conditions. It contains three distinct catalytic β subunits—β1 (Y, *PSMB6*), β2 (Z, *PSMB7*), and β5 (X, *PSMB5*)—which cleave peptide bonds after acidic, basic, and hydrophobic residues, respectively. The immunoproteasome is a specialized form of the proteasome found predominantly in immune cells, such as lymphocytes and antigen-presenting cells [5]. In these cells, the immunoproteasome primarily degrades antigens, yielding short polypeptides that are presented to the MHC-I pathway. The immunoproteasome has three active sites, including β1i (LMP2, low molecular weight protein 2, PSMB9), β2i (MECL1, multi-catalytic endopeptidase complex-like 1, PSMB10), and β5i (LMP7, low molecular weight protein 7, PSMB8).

Proteasome inhibitors profoundly affect protein homeostasis, leading to increased polyubiquitinated proteins and decreased free ubiquitin levels [6]. The two prominently ubiquitinated histones, H2A monoubiquitinated at K119 (H2AK119ub) and H2B monoubiquitinated at K120 (H2BK120ub), are deubiquitinated when cells are treated with proteasome inhibitors [7,8].

H2AK119ub is typically 5–15-fold more abundant than H2BK120ub in mammalian cell chromatin [9]. These two ubiquitinated histones have distinct chromatin locations: H2AK119ub is associated with silenced and transcriptionally active genes in heterochromatin and euchromatin, respectively, whereas H2BK120ub is associated with transcriptionally active genes in euchromatin [4,10,11].

The canonical Polycomb repressive complex 1 (PRC1) and non-canonical PRC1.1 complex (the writers) catalyze the formation of H2AK119ub [4], while removal of ubiquitin from H2AK119ub is catalyzed by deubiquitinases (USP16, the erasers) [10,12]. H2AK119ub plays a significant role in gene silencing and gene expression. Proteins and enzymes that bind to the H2AK119ub-nucleosome (the readers) include Jumonji and AT-Rich Interaction Domain 2 (JARID2), a component of the Polycomb repressive complex 2 (PRC2) complex, and DNA methyltransferase 3A (DNMT3A). Together, these readers reinforce the gene’s silenced state [12]. In contrast to the canonical PRC1 complex, the non-canonical PRC1.1 targets active genes [4].

H2BK120ub plays a major role in transcription elongation. Ubiquitination of H2BK120 is catalyzed by the Ring Finger 20/40 (RNF20/40) (the writer), which is recruited to the elongating form of RNA polymerase II (RNAP II) by the WW-containing adaptor protein with a coiled coil. Ubiquitin is removed from H2BK120ub by ubiquitin-specific peptidase enzymes (the eraser), such as USP22. This cycle of rapid ubiquitination and deubiquitination of H2BK120ub results in chromatin decondensation and compaction, thereby aiding elongation and transcription fidelity. H2BK120ub destabilizes the nucleosome, recruiting FACT, which facilitates H2A:H2B dimer removal and aids RNAP II passage [11,13,14]. In addition to FACT, several other proteins involved in elongation and chromatin modification associate with H2BK120ub-nucleosome [15]. The DOT1L’s catalytic activity to methylate H3K79 is stimulated when bound to the H2BK120ub-nucleosome [16]. H2BK120ub also enhances the noncatalytic nucleosome destabilizing activity of DOT1L [17].

In this study, we determined the effects of the proteasome inhibitor bortezomib and the immunoproteasome inhibitor ONX-0914 (PR-957) on H2BK120ub and H2AK119ub in the human leukemic cell lines MOLM-13, in which the immunoproteasome is active, and K562, in which it is not. We demonstrate that MOLM-13 cells, which express the KMT2A-MLLT3 fusion protein, have a greater level of H2BK120ub and H3K79me2 than K562 cells, which do not express KMT2A-MLLT3, consistent with the increased rate of elongation in KMT2A-MLLT3 MLL cells. Bortezomib, but not ONX-0914, reduced H2BK120ub and H2AK119ub levels in K562 and MOLM-13 cells. In MOLM-13 cells, bortezomib reduced H3K79me2 and the mRNA levels of *HOXA9*, *DOT1L*, *MYC*, and *MEIS1*.

## 2. Results

### 2.1. PTM Profiling of K562 and MOLM-13 Histones

MOLM-13, but not K562 cells, express the oncofusion protein KMT2A-MLLT3, which alters multiple epigenetic processes (e.g., the recruitment and targeting of the DOT1L enzyme) and deregulates the expression of genes (*HOXA9*) involved in MLL. Before assessing the effects of proteasome inhibition, we first compared untreated K562 and MOLM-13 cells to determine whether they had different basal levels of histone PTMs. The PTMs examined included histone H3 trimethylated at lysine 4 (H3K4me3), H2AK119ub, H2BK120ub, H3K79me2, and histone H3 trimethylated at lysine 36 (H3K36me3), which are associated with gene activation, gene silencing, and transcription elongation. Histones isolated from K562 and MOLM-13 cells were initially resolved by SDS-PAGE and stained with Coomassie Blue to determine their concentrations. The amount of histones was then adjusted to achieve equal histone loading per lane in comparative immunoblot analyses of histone PTMs. Ponceau S staining of the immunoblot before immunostaining showed the amount of histone in each sample.

**Figure 1:**
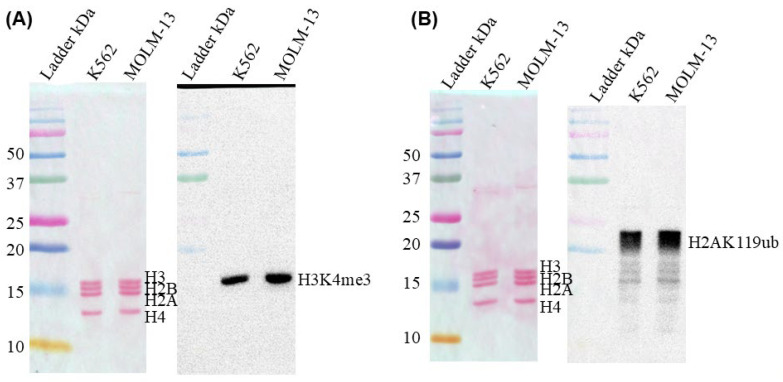
Relative abundance of H3K4me3 and H2AK119ub in K562 and MOLM-13 cells. The Ponceau S-stained blot of histones resolved by SDS 15% PAGE (**left panels**) and the accompanying blot immunostained with antibodies against H3K4me3 (**A**) and H2AK119ub (**B**) are shown (**right panels**).

The relative abundance of the active mark, H3K4me3, and the active/silent mark, H2AK119ub, in K562 and MOLM-13 cells was determined by immunoblotting. H3K4me3 is a histone PTM located on either side of the transcription start site of expressed genes. Although H2AK119ub is associated with gene silencing, it is also found in transcriptionally active chromatin. Figure 1 shows that the abundance of H3K4me3 and H2AK119ub was similar in K562 and MOLM-13 cells. Biological repeats (two for H3K4me3 and two for H2AK119ub) of the immunoblotting studies are shown in Supplementary Materials Figures S1 and S2.

**Figure 2:**
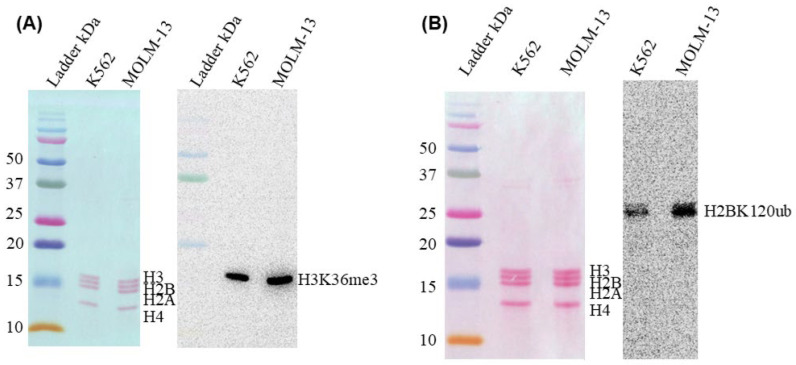
Relative abundance of H3K36me3 and H2BK120ub in K562 and MOLM-13 cells. The Ponceau S-stained blot of histones resolved by SDS 15% PAGE (**left panels**) and the accompanying blot immunostained with antibodies against H3K36me3 (**A**) and H2BK120ub (**B**) are shown (**right panels**).

**Figure 3:**
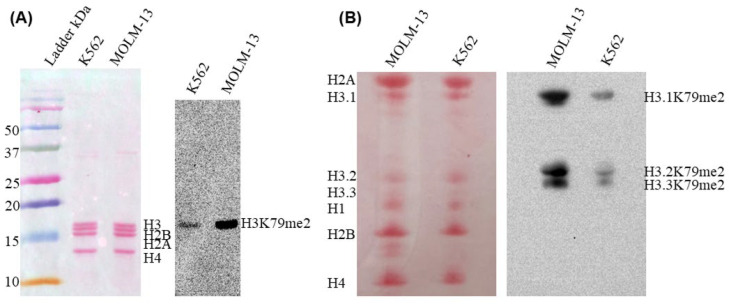
Relative abundance of H3K79me2 in K562 and MOLM-13 cells. (**A**). The Ponceau S-stained blot of histones resolved by SDS 15% PAGE (**left panel**) and the accompanying blot immunostained with antibodies against H3K79me2 are shown (**right panel**). (**B**) The Ponceau S-stained blot of histones resolved by AUT 15% PAGE (**left panel**) and the accompanying blot immunostained with antibodies against H3K79me2 (**right panel**) are shown.

Histone PTMs associated with transcription elongation include H2BK120ub, H3K79me2, and H3K36me3. The abundance of H3K36me3 was similar in K562 and MOLM-13 cells (Figure 2A, Supplementary Materials Figure S3, three biological repeats). In contrast, the abundance of H2BK120ub (Figure 2B) and H3K79me2 (Figure 3A) was higher in MOLM-13 than in K562 cells (three biological repeats shown in Supplementary Materials Figures S4 and S5A).

Histone H3 has three variants, H3.1, H3.2 and H3.3. The H3 variants were electrophoretically resolved on acetic acid-urea-Triton X-100 gels, which separate histones by size, charge, and hydrophobicity [18]. All three H3 variants were dimethylated at K79 in K562 and MOLM-13 cells, with the abundance of the K79me2 H3 variants being higher in MOLM-13 cells (Figure 3B, three biological repeats shown in Supplementary Materials Figure S5B).

**Figure 4:**
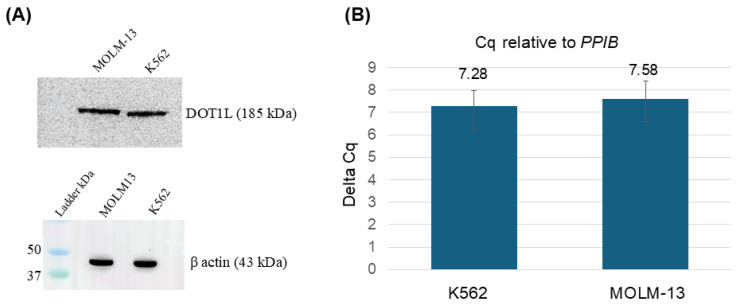
Relative expression of DOT1L in MOLM-13 and K562 cells. (**A**) Total cellular protein was separated by SDS-PAGE at 8% for DOT1L and 15% for β-actin. The immunoblots for DOT1L and β-actin are shown. (**B**) Relative transcript levels of *DOT1L* in K562 and MOLM-13 cell lines treated with 0.0005% dimethyl sulfoxide (DMSO), normalized to *PPIB*. The error bars represent the standard error of the mean (SEM) (5 biological repeats).

DOT1L (KMT4) is the only lysine methyltransferase known to methylate H3K79. To determine whether the increased abundance of H3K79me2 in MOLM-13 cells compared with K562 cells was due to elevated DOT1L levels, we measured DOT1L transcript and protein levels (Figure 4). Both the protein and transcript levels of *DOT1L* were similar in the two cell lines. Figure 4A shows that DOT1L protein levels were similar in K562 and MOLM-13 cell lysates, with β-actin as the loading control. It should be noted that, in measuring DOT1L protein levels, we obtained only one successful immunoblot. Other attempts were compromised by non-specific DOT1L antibodies and by DOT1L protein degradation. RT-qPCR results showed that *DOT1L* transcript levels, normalized to *PPIB*, were similar.

In summary, MOLM-13 and K562 cells differ in their levels of elongation-related histone PTMs, H2BK120ub and H3K79me2, with MOLM-13 cells exhibiting higher levels of both. Although the expression of DOT1L was similar in K562 and MOLM-13 cells, the increased abundance of H3K79me2 in MOLM-13 cells suggested that DOT1L’s enzymatic activity was enhanced and/or genomic targeting of DOT1L was altered in MOLM-13 cells as a consequence of the increased levels of H2BK120ub [16]. However, this conclusion should be interpreted with caution as there were issues with measuring DOT1L protein levels.

### 2.2. Sensitivity of Leukemic Cell Lines to Bortezomib

**Figure 5:**
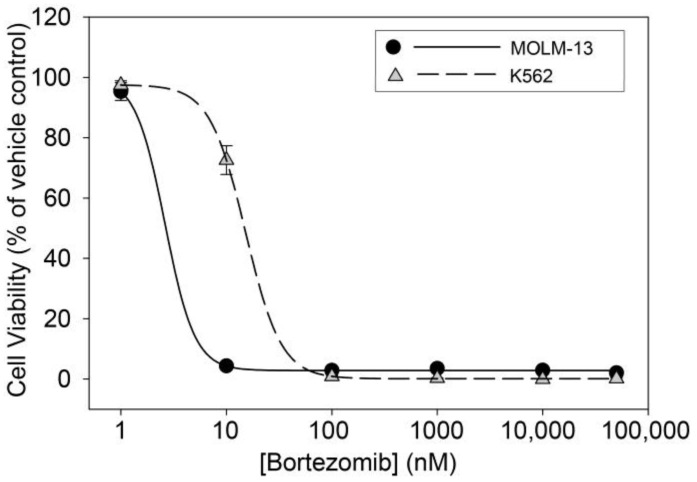
Shown are the cell viability curves for MOLM-13 (filled circles) and K562 (gray triangles) cells treated with bortezomib. Cell viability was calculated relative to the vehicle-treated control, which was set to 100%. Points represent means, and error bars indicate SD for three replicate wells per treatment condition. In most cases, the error bars are smaller than the point size and are therefore not visible. Data were fit to a 4-Parameter logistic regression using SigmaPlot 14.5 with the parameters min, max, Hill slope (n_H_) and IC_50_ listed in Table 1.

The viability of K562 (CML) and MOLM-13 (AML) cells was assessed after 72 h of treatment with bortezomib at varying concentrations. Figure 5 shows that K562 and MOLM-13 cells were sensitive to bortezomib inhibition. Fifty percent growth inhibition (IC_50_) of K562 and MOLM-13 cells was observed at concentrations of 15.2 and 2.78 nM, respectively (Table 1).

### 2.3. Effect of Bortezomib on Histone PTMs

**Figure 6:**
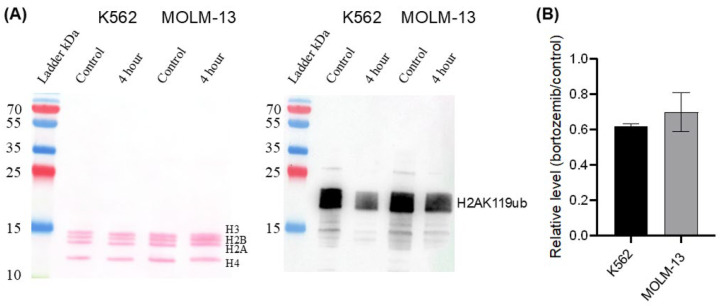
Effect of bortezomib on H2AK119ub levels in K562 and MOLM-13 cells. The cells were treated with 5 nM bortezomib or vehicle (DMSO) for 4 h. (**A**) Ponceau S-stained immunoblot of acid-extracted histones (**left panel**) and immunostained blot with anti-H2AK119ub antibody. (**B**) Quantification of H2AK119ub following bortezomib treatment of K562 and MOLM-13 cells. The error bars represent the standard error of the mean (SEM) (3 biological repeats).

Previously, we reported that the levels of ubiquitinated histones (H2Aub; H2Bub) were diminished in SKBr3 human breast tumor cells treated with the proteasome inhibitor N-acetyl-leucyl-leucyl-norlucinal (100 µM) for 4 h [7]. We therefore treated K562 and MOLM-13 cells with 5 nM bortezomib for 4 h (the same conditions used by Kamens et al. [8] to inhibit proteasome activity in acute lymphoblastic leukemia (ALL) cells), and H2AK119ub immunoblot analysis was performed on the isolated histones. A decrease in H2AK119ub after 4 h of bortezomib treatment in both cell lines was more noticeable than at 2 h (Figure 6, Supplementary Materials Figure S6, three biological repeats). In bortezomib-treated K562 and MOLM-13 cells, H2AK119ub levels were reduced to 62% and 70%, respectively, compared with the untreated controls.

**Figure 7:**
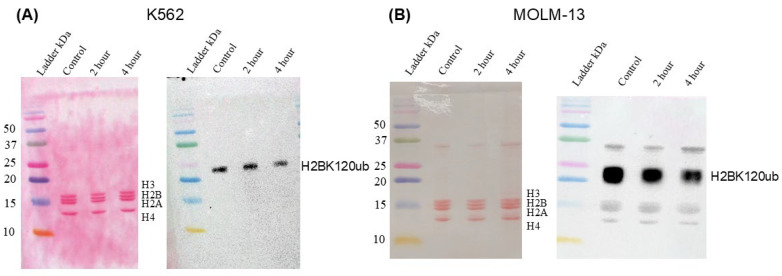
Effect of bortezomib on H2BK120ub levels in K562 and MOLM-13 cells. The cells were treated with 5 nM bortezomib for 2 and 4 h. Control-treated cells were incubated with DMSO. Ponceau S-stained immunoblot of acid-extracted histones (**left panels**) and immunostained blot with anti-H2BK120ub antibody (**right panels**). (**A**) K562, (**B**) MOLM-13. Quantification of the H2BK120ub level in MOLM-13 bortezomib (4 h) treated cells is shown in Figure 10.

H2BK120ub levels were also reduced in K562 and MOLM-13 cells after 2 and 4 h of treatment with bortezomib (Figure 7, Supplementary Materials Figure S7 (two biological repeats for K562) and Supplementary Materials Figure S8 (three biological repeats for MOLM-13)). In bortezomib-treated MOLM-13 cells, H2BK120ub levels were 47% of untreated cells (Figure 10).

**Figure 8:**
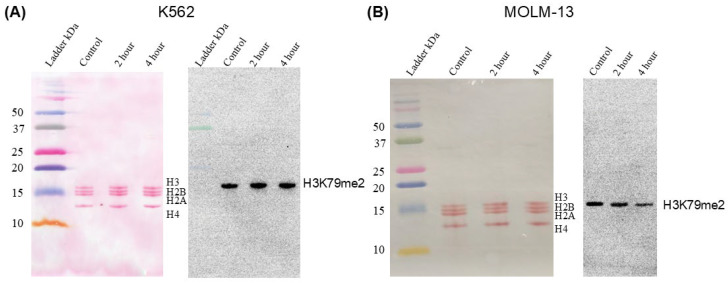
Effect of bortezomib on H3K79me2 abundance in K562 and MOLM-13 cells. The cells were treated with 5 nM bortezomib for 2 and 4 h. Control-treated cells were incubated with DMSO. Ponceau S-stained immunoblot of acid-extracted histones (**left panel**) and immunostained blot with anti-H3K79me2 antibody. (**A**) K562, (**B**) MOLM-13. Quantification of the H3K79me2 levels in MOLM-13 bortezomib (4 h) treated cells is shown in Figure 10.

Immunoblot analyses for H3K79me2 in the bortezomib-treated leukemic cells (5 nM for 2 and 4 h) showed no significant change in the K562 cells (Figure 8A, Supplementary Materials Figure S9, two biological repeats). In MOLM-13 cells, there was a slight decrease in H3K79me2 abundance at 2 h (Figure 8B, Supplementary Materials Figure S10, three biological repeats), which was more pronounced at 4 h (a 45% decline compared to untreated control; Figure 10). Electrophoretic resolution of the histones from control and bortezomib-treated (5 nM, 4 h) cells by AUT 15% PAGE showed that the intensity of the three H3 variants dimethylated at K79 decreased in the treated cells (Supplementary Materials Figure S11, three biological repeats).

**Figure 9:**
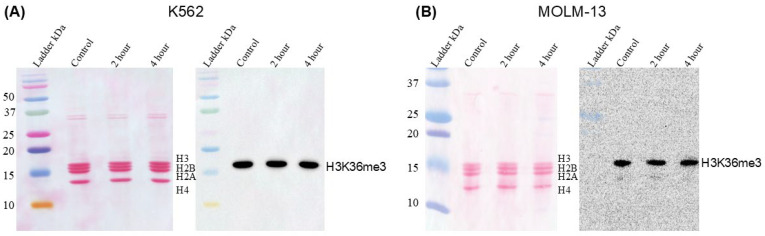
Effect of bortezomib on H3K36me3 abundance in K562 and MOLM-13 cells. The cells were treated with 5 nM bortezomib for 2 and 4 h. Control-treated cells were incubated with DMSO. Ponceau S-stained immunoblot of acid-extracted histones (**left panel**) and immunostained blot with anti-H3K36me3 antibody. (**A**) K562, (**B**) MOLM-13. Quantification of the H3K36me3 levels in MOLM-13 bortezomib (4 h) treated cells is shown in Figure 10).

Immunoblot analyses of H3K36me3 isolated from K562 and MOLM-13 cells treated with 5 nM bortezomib for 2 and 4 h revealed no changes (Figure 9; Supplementary Materials Figure S12, two biological repeats for K562, and Supplementary Materials Figure S13, three biological repeats for MOLM-13).

**Figure 10:**
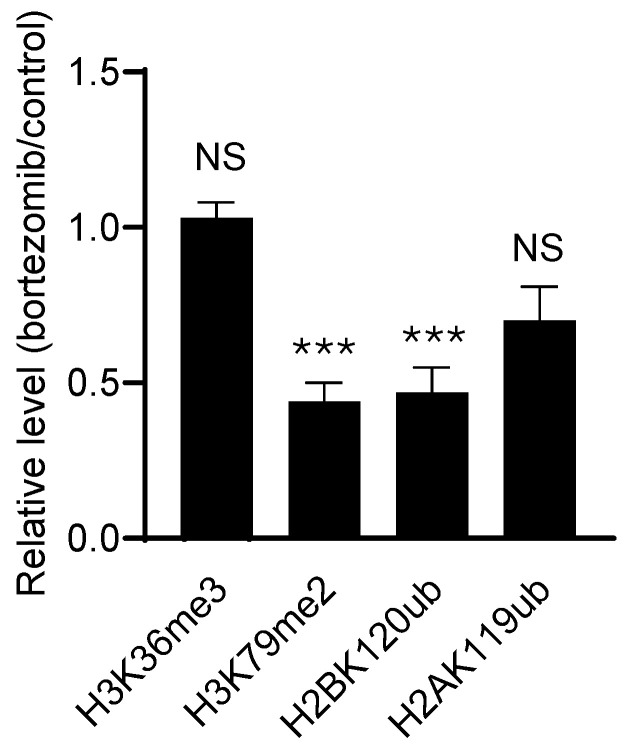
Effect of bortezomib on H3K36me3, H3K79me2, H2BK120ub and H2AK119ub in MOLM-13 cells. MOLM-13 cells were treated with 5 nM bortezomib for 4 h. The error bars represent the standard error of the mean (SEM) (3 biological repeats). The *p* values (unpaired *t*-test) are shown (NS, not significant; ***, *p* < 0.001).

In summary, 5 nM bortezomib treatment reduced the abundance of ubiquitinated histones H2A and H2B in K562 and MOLM-13 cells (Figure 10). H3K79me2 abundance was reduced in bortezomib-treated MOLM-13 cells. No change in H3K36me3 abundance was observed in either cell line.

### 2.4. Effect of Immunoproteasome Inhibitor ONX-0914 on H2BK120ub and H2AK119ub

**Figure 11:**
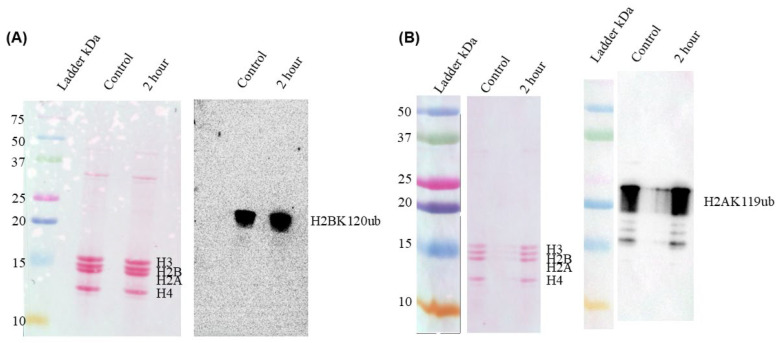
Effect of ONX-0914 on the abundance of H2BK120ub and H2AK119ub in MOLM-13 cells. The cells were treated with 50 nM ONX-0914 or vehicle control for 2 h. Ponceau S-stained immunoblot of acid-extracted histones (**left panel**) and immunostained blot with anti-H2BK120ub (**A**) and anti-H2AK119ub (**B**) antibodies.

Previous studies showed that the immunoproteasome inhibitor ONX-0914 reduced H2BK120ub levels in SEM but not RS4;11cells, even though both are ALL cell lines that express the immunoproteasome [19]. MOLM-13 cells treated with ONX-0914 (50 nM) for 2 or 4 h showed no change in H2BK120ub or H2AK119ub (Figure 11, Supplementary Materials Figures S14 and S15, three biological replicates). Even at 200 nM for 4 h, no change in H2BK120ub or H2AK119ub levels was observed in MOLM-13 or K562 cells (Supplementary Materials Figures S14B and S15).

### 2.5. Effect of Bortezomib on H2BK120ac Levels

Given the reciprocal relationship between H2BK120ub and H2BK120ac, we investigated whether loss of H2BK120ub in bortezomib-treated cells would increase H2BK120ac levels [20]. In this study, we determined the cellular levels and chromatin association of H2BK120ub and H2BK120ac in K562 and MOLM-13 cells treated with bortezomib (50 nM for 2 h). A marked decrease in H2BK120ub in the chromatin fraction of K562 and MOLM-13 cells was noted relative to the core histones when cells were treated with 50 nM bortezomib for two hours (Supplementary Materials Figure S16A, two biological repeats). The abundance of H2BK120ac was not altered in the treated cells (Supplementary Materials Figure S16B; two biological replicates).

### 2.6. Effect of Bortezomib on Transcript Levels

**Figure 12:**
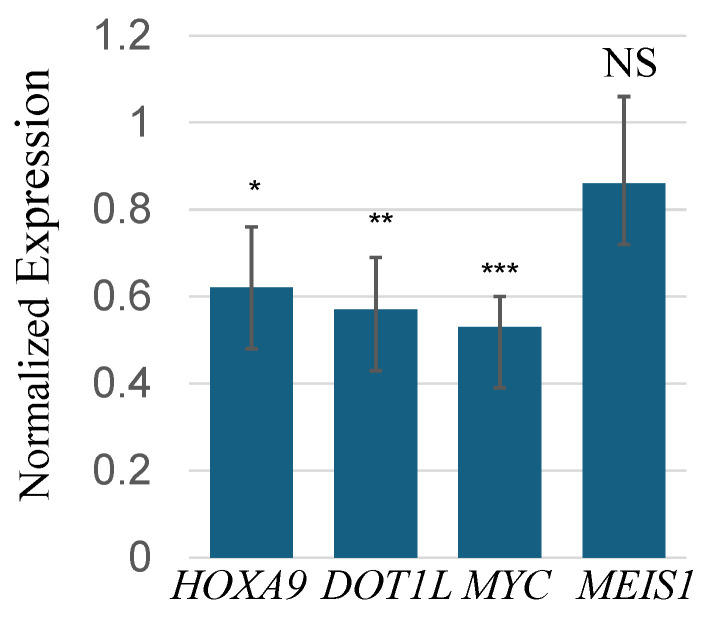
Effect of bortezomib (5 nM for 14 h) treatment of MOLM-13 cells on transcript levels of *HOXA9*, *DOT1L, MYC,* and *MEIS1*. The transcripts in the treated MOLM-13 cell lines were normalized to *PPIB*. The error bars represent the standard error of the mean (SEM) (3 biological repeats). The *p* values (unpaired *t*-test) are shown (NS, not significant; *, *p* < 0.05; **, *p* < 0.01; ***, *p* < 0.001).

MOLM-13 cells were treated with 5 nM bortezomib for 14 h, and the levels of KMT2A-MLLT3 target (*HOXA9*, *MEIS1*) and non-target genes (*DOT1L*, *MYC*) were determined (Figure 12). *HOXA9* and *MEIS1* transcript levels were reduced (0.62 and 0.86, respectively) in MOLM-13-treated cells, although in three biological repeats, the reduction in *MEIS1* in the treated cells failed to reach statistical significance. The transcript levels of *MYC* and *DOT1L* fell lower (0.53 and 0.57, respectively) than the KMT2A-MLLT3 target transcripts in MOLM-13-treated cells.

## 3. Discussion

The distribution of nucleosomes with histone PTMs associated with transcribed genes follows the order: H3K4me3 nucleosomes on either side of the transcription start site, followed by H2BK120ub and H3K79me2 along the gene body, and finally H3K36me3 along the gene body [21,22,23]. H3K4me3 levels were similar in K562 and MOLM-13 cells. In contrast, the elongation marks H2BK120ub and H3K79me2 were higher in MOLM-13 cells than in K562 cells, whereas the levels of H3K36me3 and DOT1L were similar. Measuring DOT1L protein levels in K562 and MOLM-13 was problematic in our immunoblot analyses due to the limited availability of DOT1L-specific antibodies. We tested anti-DOT1L antibodies from three different vendors. Unfortunately, they all had non-specific binding.

The increased levels of the H2BK120ub and H3K79me2 in MOLM-13 cells could be explained by the KMT2A-MLLT3 super-elongation complex associated with the elongating form of RNAP II [24,25]. Expression of KMT2A-MLLT3 enhances the rate of transcriptional elongation in MOLM-13 AML cells. The ubiquitination of H2BK120 depends on ongoing elongation, and DOT1L’s activity to methylate H3K79 is stimulated upon binding to the H2BK120ub-modified nucleosome [26]. The higher basal levels of H2BK120ub and H3K79me2 in MOLM-13 cells suggest enhanced transcription elongation-associated chromatin activity in the KMT2A-MLLT3-rearranged cell line. This relationship is consistent with the known role of H2BK120ub in supporting DOT1L-mediated H3K79 methylation. Following bortezomib treatment, the reduction in H2BK120ub, together with the decrease in H3K79me2 in MOLM-13 cells, suggests that proteasome inhibition disrupts this H2BK120ub-H3K79me2 elongation-associated axis. In contrast, the lack of change in H3K36me3 indicates that bortezomib does not broadly reduce all elongation-associated histone PTMs in the short term (four hours). These findings support a model in which bortezomib interferes with chromatin features that contribute to transcriptional elongation and MLL-associated gene expression in MOLM-13 cells.

There are several possible explanations for why H3K36me3 was not increased in MOLM-13 cells. The main enzyme that tri-methylates H3K36 is SETD2, which is associated with the elongating RNAP II apparatus [27,28]. The protein complexes (and/or their enzymatic activities) bound to the RNAP II complex may change as RNAP II travels along the gene body. There is crosstalk between H3K36me3 and H3K79me2 via SETD2, with a loss of SETD2 activity resulting in decreased H3K36me3, increased H3K79me2, and increased RNAP II activity [23,29]. Alternatively, the increased levels of the KDM4s (KDM4A,B,C,D), which demethylate H3K36me3, observed in cells with KMT2A-MLLT3, may mute an elongation-driven increase in H3K36me3 [30]. For example, increased KDM5B activity in MCF7 breast cancer cells lowers steady-state levels of H3K4me3 relative to those in MDA-MB-231 breast cancer cells with lower KDM5B expression. Further studies will be required to explain why some elongation-related histone PTMs show increased levels, while others do not. Unlike H2BK120ub, H2AK119ub is not dependent upon ongoing transcription [31]. The steady-state level of H2AK119ub was similar in K562 and MOLM-13 cells.

The level of H2BK120ub is often lower in cancer cells than in their normal counterparts [32,33]. However, genes involved in cancer development show elevated levels of H2BK120ub across their gene bodies. Our results suggest that, unlike other cancers, H2BK120ub levels in KMT2a-rearranged leukemia (AML, ALL) will be higher than in their normal hematopoietic counterparts. To the best of our knowledge, this remains undetermined. This hypothesis will require further experimental validation.

Previous studies have demonstrated the sensitivity of AML and ALL cells to the proteasome inhibitor bortezomib [8]. In our study, to maintain experimental consistency, the same predefined broad concentration range was used for all tested inhibitors and both cell lines in the initial dose–response experiments. This design was intended to capture the full response range across conditions; however, concentrations substantially above the apparent plateau provide limited additional information for IC_50_ estimation, and a denser concentration series around the low-nanomolar transition region would improve the precision of cell line-specific estimates. Nevertheless, the observed IC_50_ values were broadly consistent with published bortezomib sensitivity data: the K562 IC_50_ of 15.2 nM was close to the 20 nM value reported in a 72 h MTT assay [34], whereas the MOLM-13 IC_50_ of 2.78 nM was within the low-nanomolar range previously reported after 72 h (6.8 ± 3.6 nM [35]) and after 24 h (7.9 nM in wild-type MOLM-13 cells [36]).

In immunoblot analyses, Kamens et al. reported that 50 nM bortezomib treatment reduced H2BK120ub levels in ALL cells after 30–120 min of exposure [8]. Both K562 and MOLM-13 showed reduced levels of H2BK120ub and H2AK119ub after 4 h of treatment with 5 nM bortezomib and after 2 h at 50 nM. Both ubiquitinated histones have rapid turnovers (t½ 90 min for H2AK119ub) [37]. Among the enzymes that deubiquitinate ubiquitinated histones are ubiquitin-specific proteases (USPs), which deubiquitinate H2AK119ub and H2BK120ub (e.g., USP3, USP7, USP22) [38,39,40]. Proteasome inhibition reduces free ubiquitin levels, thereby increasing polyubiquitination of proteins. Ubiquitinated histones are “sacrificed” to serve the high demand for free ubiquitin. The action of the histone deubiquitinating enzymes releases free ubiquitin, which is used to polyubiquitinate specific proteins [7].

Bortezomib inhibits the canonical and immunoproteasomes. Inhibition of the immunoproteasome did not decrease the steady-state levels of H2BK120ub and H2AK119ub in ONX-0914-treated MOLM-13 cells. This result suggests that in MOLM-13 cells, the canonical proteasome’s activity exceeds that of the immunoproteasome in the processing and degradation of polyubiquitinated proteins. In cases in which immunoproteasome inhibition results in decreased H2BK120ub levels in SEM (ALL) cells, the immunoproteasome activity may exceed that of the canonical proteasome [19].

Within hours following the addition of bortezomib to leukemic cells, the loss of H2BK120ub and perhaps H2AK119ub will hinder transcription elongation [7,38,41]. A noticeable decrease in transcription was reported after four hours following proteasome inhibition [7]. In measuring specific cellular transcript levels, the reduction in these levels following transcription inhibition would depend, in part, on the transcript’s stability. Following treatment of MOLM-13 cells with 5 nM bortezomib for 14 h, the transcript levels of *MYC*, *DOT1L*, and *HOXA9* were reduced. *MYC* transcripts, which have a rapid turnover rate, showed the largest reproducible decrease in bortezomib-treated MOLM-13 cells [42].

The treatment of KMT2a-rearranged leukemic cells with bortezomib and the reduction in H2BK120ub and H2AK119ub would affect several epigenetic events deregulated in leukemia, thereby altering gene expression (e.g., *HOXA9*) that drives the leukemic state. The loss of H2BK120ub will attenuate DOT1L-mediated H3K79me2, while the loss of H2AK119ub will impact the PRC1 pathways [4,10,43,44]. A phase 3 trial found that bortezomib, when combined with standard therapy, did not improve treatment outcomes in pediatric AML patients [45]. However, a reverse-phase protein array-based proteomic study of peripheral blood samples from AML patients reported that patients with high HME (16 histone- and chromatin-modifying enzymes) expression had significantly improved survival when treated with bortezomib-containing chemotherapy [46]. The HME included the histone modifying enzymes KDM1A, ASH2L, HDAC6, HDAC3, HDAC1, JMJD6, SIRT6, HDAC2, and SIRT1. Given that several of the histone-modifying enzymes identified belong to the histone deacetylases (HDACs) family, it is noteworthy that combined treatment with bortezomib and the HDAC inhibitor vorinostat has been reported to improve clinical responses in patients with AML and ALL [8]. Of note, vorinostat potently reduces KDM1A expression [47]. Also, the DOT1L inhibitor EPZ5676 reduces KDM1A and HOXA9 [48]. The inclusion of histone-modifying enzymes involved in KMT2a-rearranged leukemia (e.g., RNF20, BCOR (PRC1.1 complex), KDM4s) may help further identify AML patients’ response to bortezomib-containing therapies.

## 4. Materials and Methods

### 4.1. Cell Lines and Cell Culture

The human leukemic cell lines MOLM-13 (acute myeloid leukemia) and K562 (chronic myeloid leukemia) were used in this study. MOLM-13 cells were obtained from DSMZ (Leibniz Institute—DSMZ German Collection of Microorganisms and Cell Cultures GmbH, Stöckheim-Leiferde, Germany), and K562 cells were obtained from ATCC (ATCC.ORG, American Type Culture Collection, Manassas, VA, USA). Both cell lines were cultured in Roswell Park Memorial Institute-1640 (RPMI-1640) medium (Thermo Fisher Scientific, Whitby, ON, Canada, #MT10041CV) supplemented with 10% fetal bovine serum (FBS, Thermo Fisher Scientific, Whitby, ON, Canada, #MT35077CV) and 1% penicillin-streptomycin (Thermo Fisher Scientific, Whitby, ON, Canada, #15140122) in 175 cm^2^ tissue culture flasks (Thermo Fisher Scientific, Whitby, ON, Canada, #10-126-13) at 37 °C in a humidified atmosphere of 5% CO_2_. The cells were subcultured when they reached 80–90% confluency. Trypan Blue stain (Thermo Scientific, Burlington, ON, Canada) was used for cell counting and cell viability assessment.

### 4.2. MTS Assay for Inhibitor Dose–Response Studies

MOLM-13 and K562 cells were seeded into 96-well plates with 90 µL of medium containing 1.1 × 10^5^ cells/mL per well. Subsequently, 10 µL of medium containing bortezomib was added to achieve final concentrations ranging from 0 to 50 µM in a final volume of 100 µL per well, while ensuring the final DMSO concentration did not exceed 1%. To maintain experimental consistency, the same predefined broad concentration range was applied to all tested inhibitors and both cell lines as part of the initial dose–response screening design. After 72 h, 10 µL of MTS reagent (CellTiter 96^®^ AQueous One Solution Cell Proliferation Assay; Promega, Madison, WI, USA) was added to each well, and the plates were incubated for an additional 3 h. For each cell line and treatment condition, three separate wells were seeded and treated in parallel. After incubation, the absorbance of the samples was measured at 490 nm (Synergy H1 microplate reader, BioTek Co., Winooski, VT, USA) to assess cell viability, expressed as a percentage of the control group. The data for each cell line and drug were fit to a four-parameter logistic regression model, and the IC_50_ values for each inhibitor were derived using SigmaPlot 14.5.

### 4.3. Bortezomib and ONX-0914 Treatments

Ten mM bortezomib (MilliporeSigma Canada Ltd., Oakville, ON, Canada, #504314) and selective PSMB8 inhibitor ONX-0914 (Cayman Chemical, Ann Arbor, MI, USA, #16271) were prepared by reconstitution in DMSO (MilliporeSigma Canada Ltd., Oakville, ON, Canada, #D4540). Bortezomib and ONX-0914 at 10 μM in 0.1% DMSO was prepared by diluting the 10 mM stock in PBS (137 mM NaCl, 2.7 mM KCl, 10 mM Na_2_HPO_4_, 1.8 mM KH_2_PO_4_, pH 7.4). K562 and MOLM-13 cells at 70–90% confluency were treated with 5 or 50 nM bortezomib in 0.00005% or 0.0005% DMSO, respectively and incubated for 2, 4, or 14 h under the same conditions as described above. DMSO (0.1%) was used as the vehicle for the control conditions. K562 and MOLM-13 cells at 70–90% confluency were alternatively treated with 50 nM ONX-0914 in 0.00005% DMSO or 200 nM ONX-0914 in 0.02% DMSO and incubated for 2 or 4 h, respectively, under the same conditions as described above. These concentrations were chosen based on previous immunoproteasome inhibition studies with leukemic cells [19,49].

### 4.4. Histone Extraction

Histones were isolated from MOLM-13 and K562 cells using two methods (H_2_SO_4_- and HCl-extraction) [50,51]. Although both methods yielded comparable results in immunoblot analyses, the HCl-extraction method produced higher histone yields. Histones were quantified using the Bradford assay.

### 4.5. Cellular Lysate Preparation

To determine DOT1L protein levels, MOLM-13 and K562 cells cultured under standard conditions were harvested for cell lysate preparation as described in [50]. The lysates were then separated by SDS-PAGE, and immunoblotting was performed to determine DOT1L levels in both cell lines using a DOT1L antibody.

### 4.6. Cell Fractionation and Total Cell Lysates

Fifty million cells were harvested post-treatment of K562 and MOLM-13 cells with fifty nM bortezomib for 2 h as described above. The cell pellet was resuspended in extraction buffer (10 mM HEPES (pH 7.9), 10 mM KCl, 1.5 mM MgCl_2_, 0.34 M sucrose, 10% (*v*/*v*) glycerol, 5 mM sodium butyrate) containing 0.2% IGEPAL, protease and phosphatase inhibitors (PPI, Thermo Scientific, Burlington, ON, Canada, Halt ™ Protease and Phosphatase Inhibitor Cocktail, #78441), added as per manufacturer’s instructions), and 2 mM PMSF. The cell suspension was incubated on ice for 10 min with occasional rotation, followed by centrifugation at 6500× *g* for 5 min at 4 °C and collection of the supernatant (cytoplasmic fraction). The pellet was resuspended in the extraction buffer containing PPI and 2 mM PMSF, incubated on ice for 1 min, then centrifuged at 6500× *g* for 5 min at 4 °C, and the supernatant collected (wash buffer supernatant). The nuclei pellet was resuspended in no-salt buffer (3 mM EDTA, 0.2 mM EGTA, 5 mM sodium butyrate, with PPI and 2 mM PMSF), mixed intermittently for 1 min (10 s on, 10 s off), and incubated for 30 min at 4 °C on a rotator. The nuclei were collected by centrifugation (6500× *g* for 5 min at 4 °C), and the supernatant (nucleoplasm fraction) was collected. The chromatin-containing pellet was resuspended in RIPA Buffer (Thermo Scientific, Burlington, ON, Canada, #89900) with PPI and 2 mM PMSF, then sonicated (Power = 2, continuous mode (3 cycles, 30 s on, 30 s off, Thermo Fisher Scientific, Whitby, ON, Canada, Model 100, Ultrasonic Dismembrator) to yield the chromatin fraction. A260 measurements were taken for all fractions.

To obtain the total cellular lysate, 25 million cells were harvested after 2 h of treatment with 50 nM bortezomib, as described above. The cell pellet was washed once with PBSB buffer (137 mM NaCl, 2.7 mM KCl, 10 mM Na_2_HPO_4_, 1.8 mM KH_2_PO_4_, 5 mM sodium butyrate, pH 7.4) containing protease and phosphatase inhibitors (Thermo Scientific, Burlington, ON, Canada, Pierce ™ Protease and Phosphatase Inhibitor Mini Tablet, #A32961, added as per manufacturer’s instructions) and 2 mM PMSF, followed by resuspension in 750 µL of RIPA Buffer (Thermo Scientific, Burlington, ON, Canada, #89900; with PPI and 2 mM PMSF) and sonication (Power = 2, continuous mode, 3 cycles, 30 s on, 30 s off, Thermo Fisher Scientific, Whitby, ON, Canada, Model 100, Ultrasonic Dismembrator) to yield the total cellular lysate.

### 4.7. SDS-PAGE and Immunoblotting

SDS-PAGE gels and immunoblots were prepared as outlined in [50]. Protein ladders (Thermo Fisher Scientific, Whitby, ON, Canada, #26619 or Bio-Rad Laboratories Ltd., Mississauga, ON, Canada, #1610395) were run alongside the samples to verify target protein detection by molecular mass. Antibodies used in this research are listed in Supplementary Data Table S1. Ponceau S staining and antibody detection were performed on the same membrane.

### 4.8. Acetic Acid-Urea-Triton-X100 Polyacrylamide Gel (AUT) Electrophoresis and Immunoblotting

Fifteen % polyacrylamide AUT (acetic acid/6 M urea/0.375% Triton X-100, Thermo Fisher Scientific, Whitby, ON, Canada) minilab gel was prepared. The acid-extracted histones were mixed with 2X AUT-sample buffer (0.75 M KAc pH 4.0, 30% (*w*/*v*) sucrose, 0.1% (*w*/*v*) pyronin Y) and loaded onto an AUT. A running buffer of 0.9 N acetic acid was used, and the gel was electrophoresed at 200 V for 2 h at room temperature (for more details see [52]). For immunoblotting, the AUT gel was equilibrated in two steps before being transferred to the nitrocellulose membrane. First, the gel was submerged in 50 mM glacial acetic acid and 0.5% SDS (equilibration buffer 1) for two 30 min incubations, followed by two 30 min incubations in a Tris-SDS buffer (equilibration buffer 2) containing 1 M Tris-HCl (pH 6.8), 2.3% SDS, and 5% β-mercaptoethanol. After equilibration, the gel was transferred to a nitrocellulose membrane using the Trans-Blot Turbo Transfer System (Bio-Rad Laboratories Ltd., Mississauga, ON, Canada). The membrane was processed as described above for SDS 15% polyacrylamide gels.

### 4.9. Total RNA Extraction and cDNA Synthesis

Total RNA extraction was performed using the RNeasy Plus Mini Kit (Qiagen, Toronto, ON, Canada, #74134) following the manufacturer’s instructions with the addition of washing the harvested cell pellet with ice-cold PBS prior to lysis with Buffer RLT Plus containing β-mercaptoethanol. Additionally, the homogenized lysate was run through a QIAshredder spin column (Qiagen, Toronto, ON, Canada, #79656) prior to transfer to a gDNA eliminator spin column to increase RNA yield. Eluted RNA concentration and purity were assessed using the A260/A280 and A260/A230 ratios with a Nanodrop 2000 (Thermo Fisher Scientific, Whitby, ON, Canada). Following RNA isolation, DNase I treatment was performed using the DNA-free™ DNA removal kit (Thermo Fisher Scientific, Whitby, ON, Canada, #AM1906) as per manufacturer’s instructions. Purification of RNA was assessed by PCR using primers designed across exons of housekeeping genes. cDNA synthesis was performed using the iScript™ Reverse Transcription Supermix for RT-qPCR (Bio-Rad Laboratories Ltd., Mississauga, ON, Canada, #1708840) according to the manufacturer’s instructions. Synthesized cDNA was stored at −20 °C.

### 4.10. Primer Design, Primer Sequencing

Gene transcripts were obtained from Ensembl (https://www.ensembl.org/, accessed on 23 January 2023 and 3 July 2025), and the transcript variant best suited to the experimental needs was selected. Once identified, the transcript sequence was retrieved, and *HOXA9*, *DOT1L* and *MEIS1* primers were designed using Integrated DNA Technologies (IDT’s) RealTime qPCR Assay Entry (https://www.idtdna.com/scitools/Applications/RealTimePCR/default.aspx, accessed on 3 July 2025) or PrimerQuest tools (https://www.idtdna.com/PrimerQuest/Home/Index, accessed on 23 January 2023). Primer specificity was confirmed using UCSC’s in silico PCR tool (https://genome.ucsc.edu/cgi-bin/hgPcr, accessed on 29 July 2025). The *MYC* primers were from [53]. Following primer design, a gradient real-time PCR was performed to confirm the absence of primer dimers for each primer, with annealing temperatures selected based on melt curve analysis. Finally, PCR products were run on 1–2% agarose gels to verify their lengths. The primer sequences used for the reverse transcriptase quantitative PCR (RT-qPCR) experiments and analysis are detailed in Supplementary Data Table S2.

### 4.11. RT-qPCR (Reverse Transcription Quantitative PCR)

For each PCR sample, a mixture was prepared by combining 10 microliters of PowerUp™ SYBR™ Green master mix (Thermo Fisher Scientific, Whitby, ON, Canada, #A25741), 1 microliter each of forward and reverse primers, 7 microliters of nuclease-free water, and 1 microliter of previously synthesized cDNA, resulting in a total reaction volume of 20 microliters. Following preparation, 96-well plates were utilized, and the QuantStudio™ thermocycler (Thermo Fisher Scientific, Whitby, ON, Canada) was employed for amplification. The PCR conditions were set as follows. The reaction mixture was denatured at 95 °C for 2 min, followed by denaturation at 95 °C for 30 s, annealing at 60 °C for 30 s, and extension at 72 °C for 1 min for 40 cycles. Subsequently, a melt curve analysis was performed by heating the samples to 95 °C for 15 s, annealing at 50 °C for 1 min, and finally denaturing at 95 °C for 15 s. The cycle threshold (Ct) values obtained were normalized to the corresponding GAPDH or PPIB expression using the ΔCt method, where higher ΔCt values indicated lower amounts of the transcript. In certain experiments, target levels were determined using the ΔΔCt method, in which ΔCt values for each cell line were normalized to an appropriate control. Fold changes were calculated using the 2^−ΔΔCt^ formula.

### 4.12. Statistical Analysis

Changes in histone post-translational modifications (PTMs) or histone variants were analyzed using the Student *t*-test. Statistical evaluations were conducted with Prism v10 software (GraphPad Software, San Diego, CA, USA). Data were presented as the mean ± standard error of the mean (SEM) from three independent experiments.

## 5. Conclusions

In summary, low concentrations of bortezomib reduced H2AK119ub, H2BK120ub and, to a lesser extent, H3K79me2 in an MLL subtype of AML cells. Transcriptional elongation is augmented in MLL. The reduction in H2BK120ub and H3K79me2 is consistent with bortezomib reducing elongation, leading to the silencing of genes (*HOXA9*) that propagate MLL. Thus, incorporating bortezomib into a therapeutic regimen for this form of MLL would be beneficial. Current studies report that profiling of histone-/chromatin-modifying enzymes aids in identifying patient response to the treatment. Further identification of histone-/chromatin-modifying enzymes that play important roles in AML subtypes could yield combinations of bortezomib and histone-modifying enzyme inhibitors better tailored to the patient’s cancer.

## Figures and Tables

**Table 1:** Fit parameters for K562 and MOLM-13 cells treated with bortezomib.

Cells	Min (%)	Max (%)	n_H_	IC_50_ (nM)
K562	0.104 ± 0.15	97.6 ± 1.5	−2.59 ± 0.28	15.2 ± 1.3
MOLM-13	2.76 ± 0.30	98.8 ± 2.2	−3.25 ± 0.35	2.78 ± 0.11

## Data Availability

The original contributions presented in this study are included in the article/Supplementary Material. Further inquiries can be directed to the corresponding author(s).

## Supplementary Materials

The following supporting information can be downloaded at: https://www.mdpi.com/article/10.3390/ijms27156597/s1.
