# Supplementary material for: Effect of the Proteasome Inhibitor, Bortezomib, on Histone Modifications in Human Leukemic Cell Lines
Source: Int J Mol Sci. 2026 Jul 24;27(15):6597. doi: 10.3390/ijms27156597 (PMC13465501; doi:10.3390/ijms27156597)
Supplement: Supplementary file 1 [file ijms-27-06597-s001.zip › ijms-4389551-supplementary.pdf]

Figure S1. Relative abundance of H3K4me3 in K562 (K) and MOLM-13 (M) cells. The Ponceau S-stained blot of histones resolved by SDS 15% PAGE (left panels), and the accompanying blot immunostained with antibodies against H3K4me3 are shown (right panels).

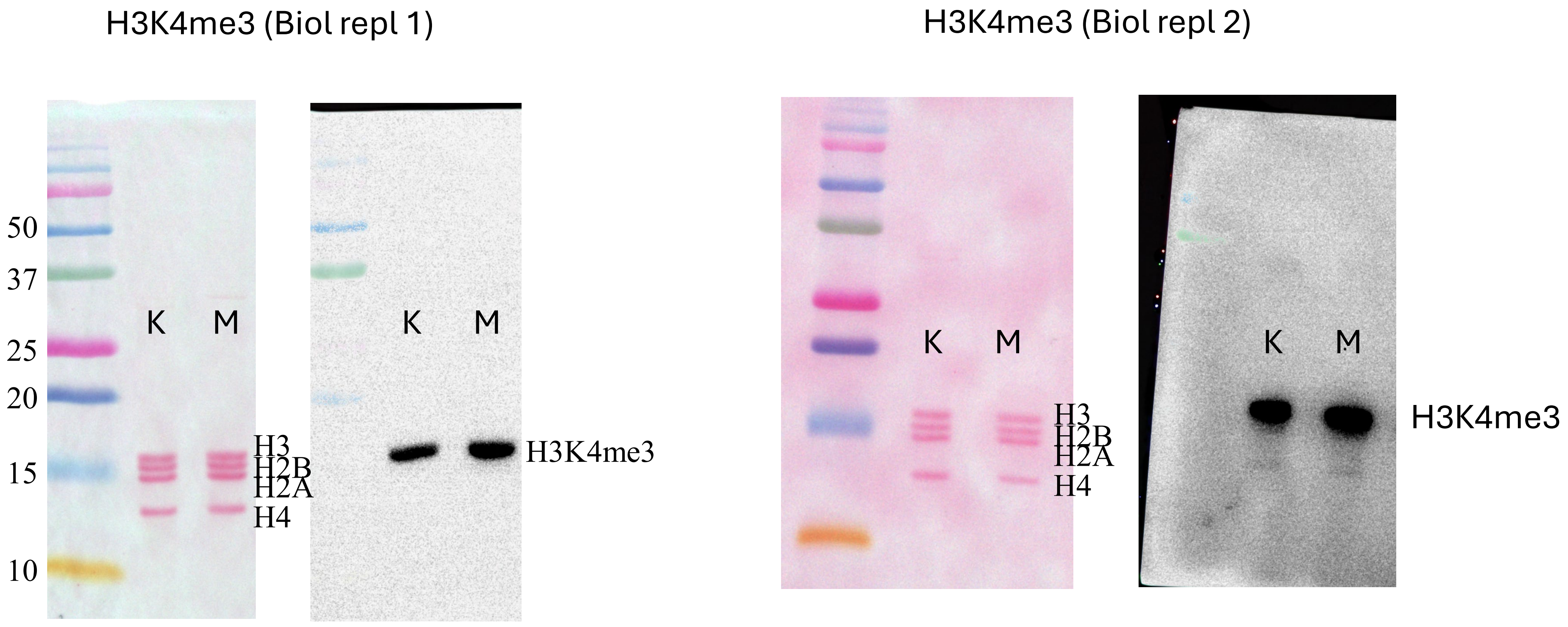

Figure S2. Relative abundance of H2AK119ub in K562 (K) and MOLM-13 (M) cells. The Ponceau S-stained blot of histones resolved by SDS 15% PAGE (left panels), and the accompanying blot immunostained with antibodies against H2AK119ub are shown (right panels).

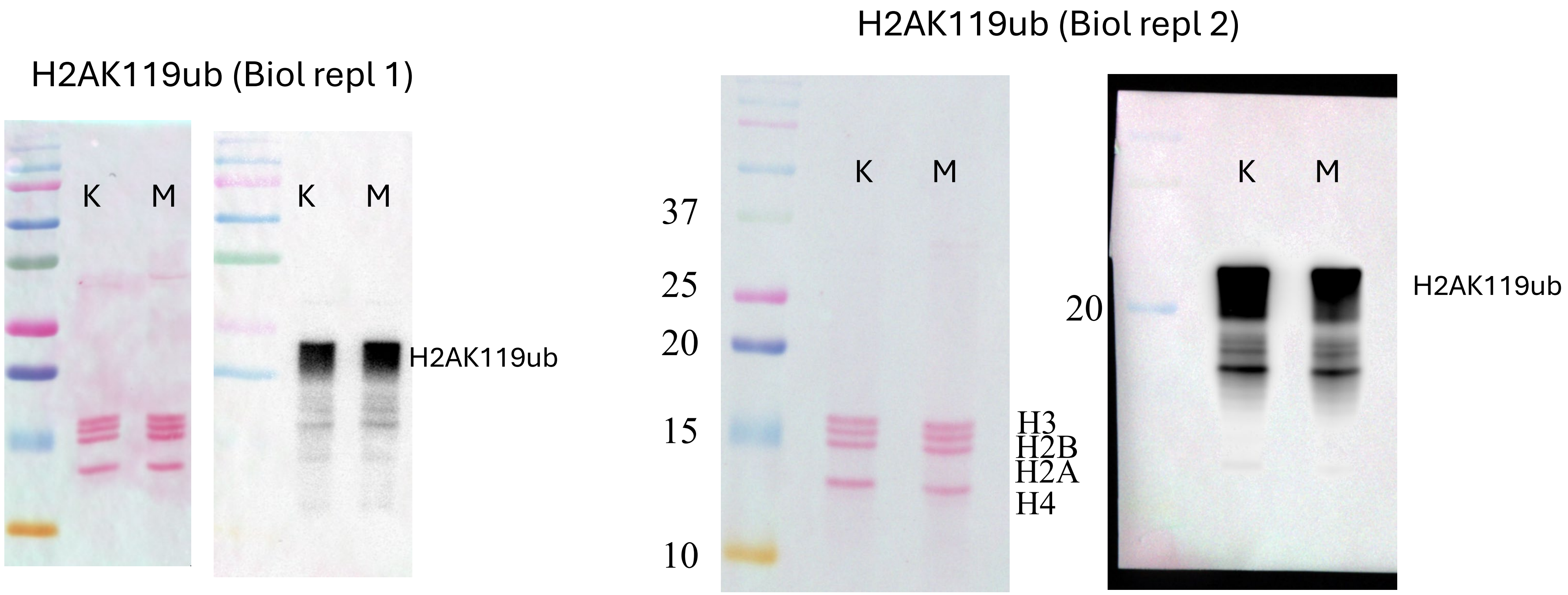

Figure S3. Relative abundance of H3K36me3 in K562 (K) and MOLM-13 (M) cells. The Ponceau S-stained blot of histones resolved by SDS 15% PAGE (left panels), and the accompanying blot immunostained with antibodies against H3K36me3 are shown (right panels).

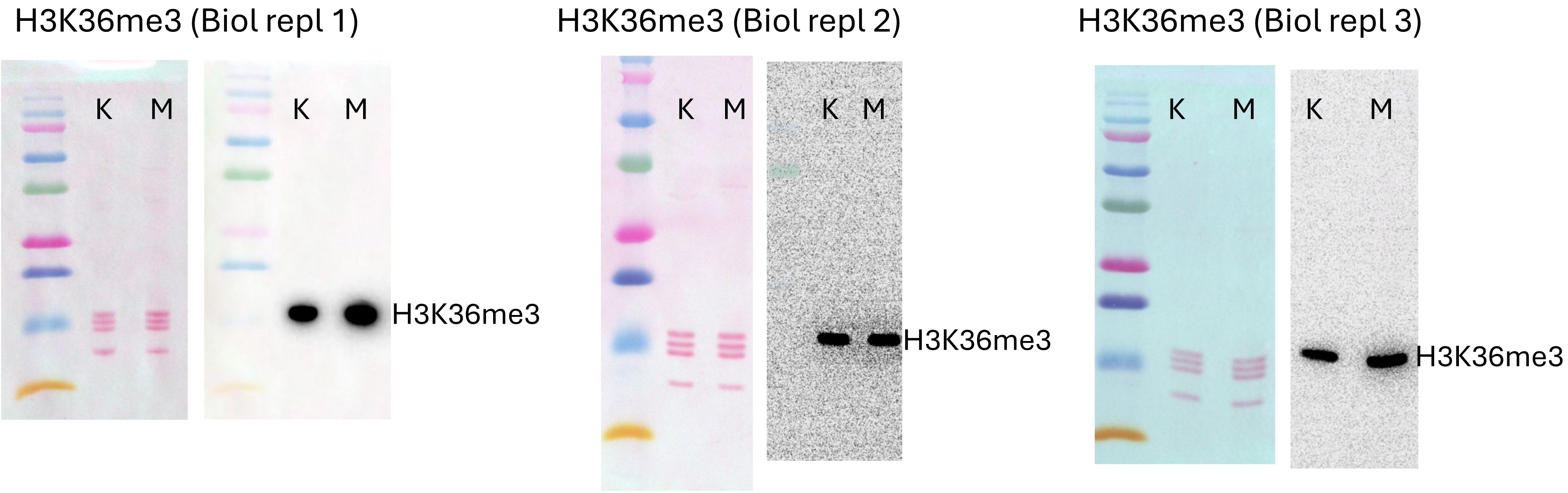

Figure S4. Relative abundance of H2BK120ub in K562 (K) and MOLM-13 (M) cells. The Ponceau S-stained blot of histones resolved by SDS 15% PAGE (left panels), and the accompanying blot immunostained with antibodies against H2BK120ub are shown (right panels).

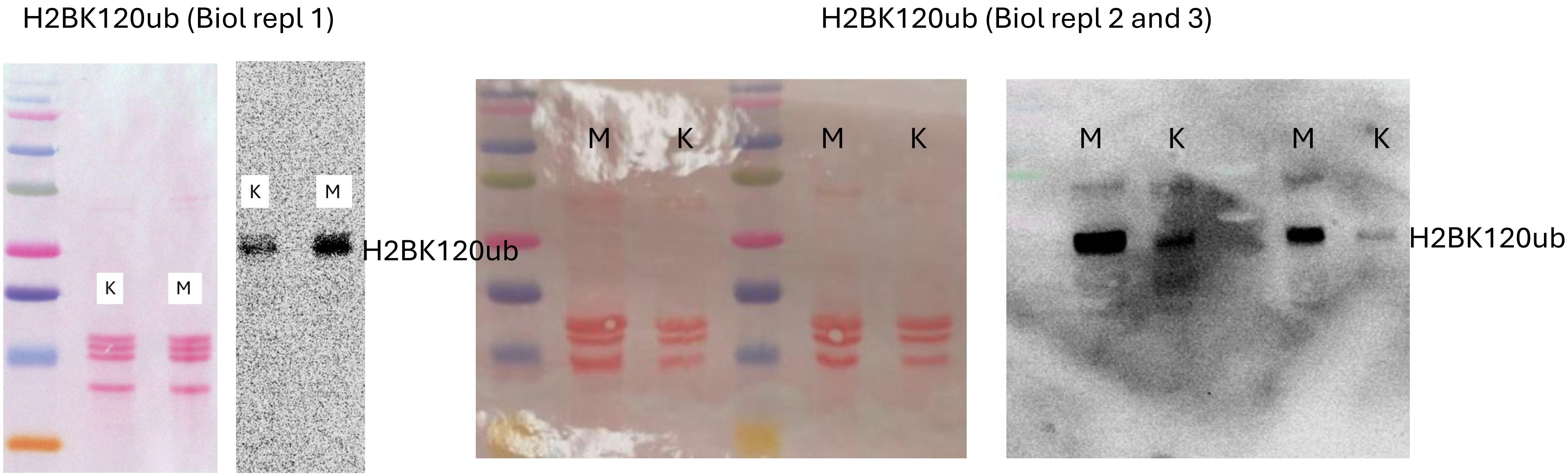

Figure S5A. Relative abundance of H3K79me2 in K562 (K) and MOLM-13 (M) cells. The Ponceau S-stained blot of histones resolved by SDS 15% PAGE (left panels), and the accompanying blot immunostained with antibodies against H3K79me2 are shown (right panels).

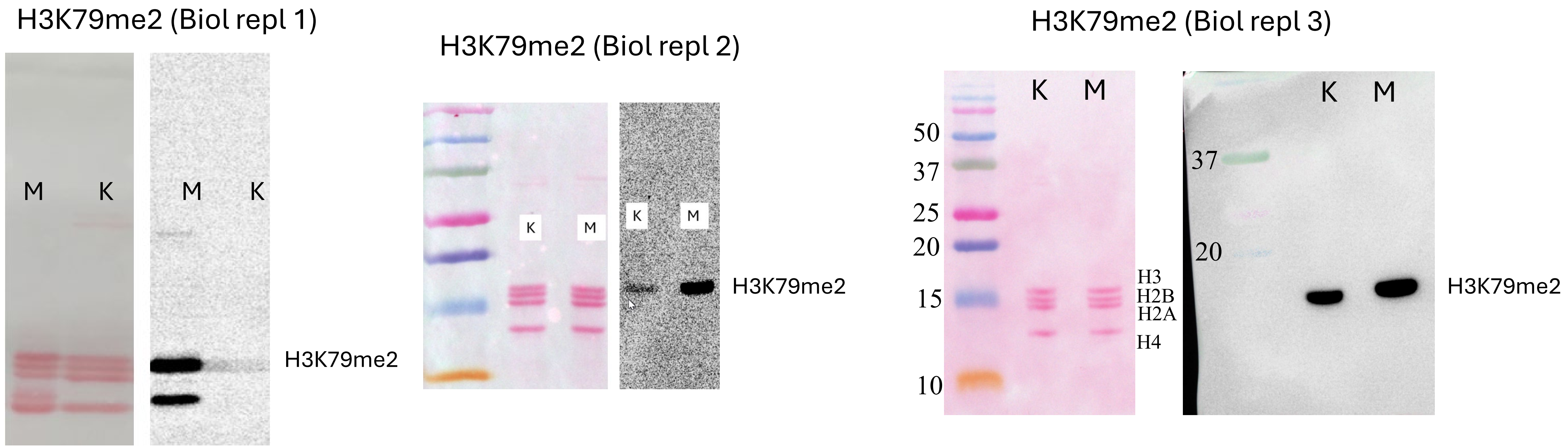

Figure S5B. Relative abundance of H3K79me2 in H3 variants in K562 (K) and MOLM-13 (M) cells. The Ponceau S-stained blot of histones resolved by AUT 15% PAGE (left panel), and the accompanying blot immunostained with antibodies against H3K79me2 (right panel) is shown.

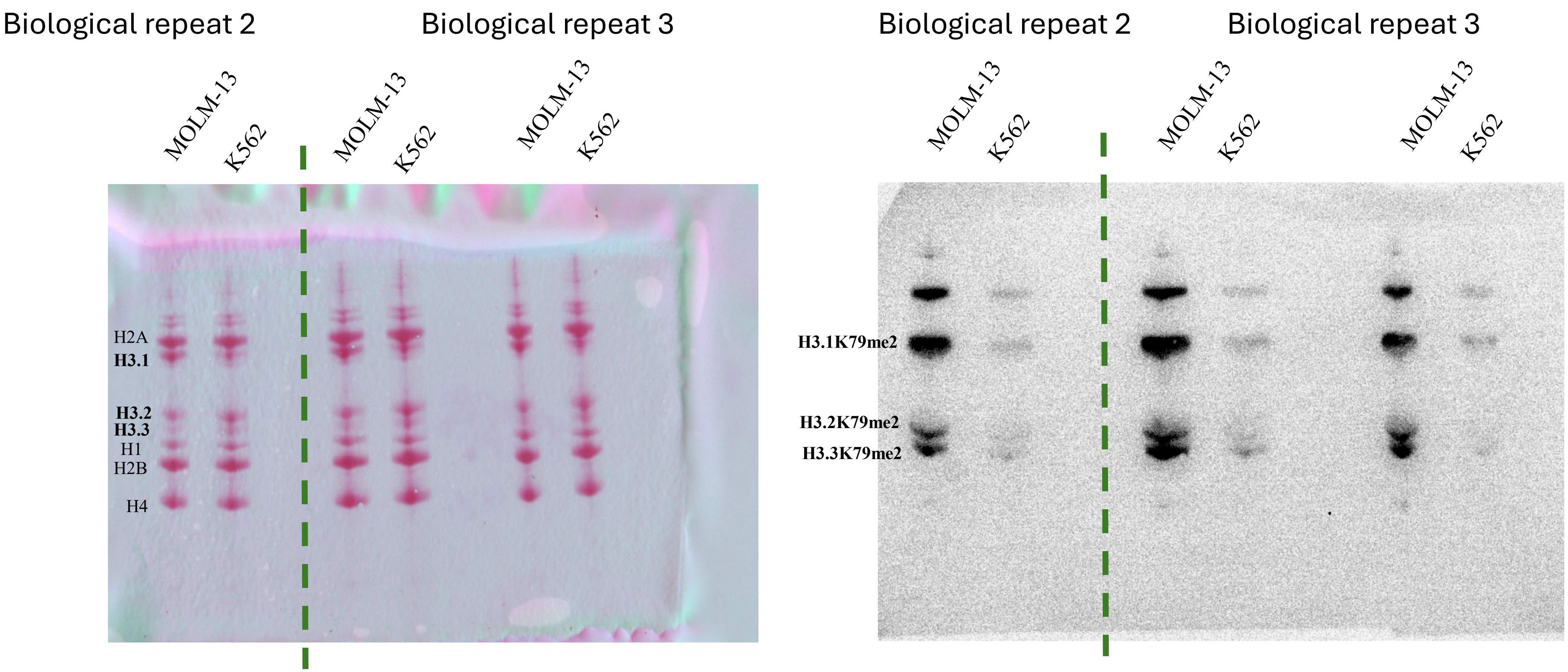

Figure S6A. Effect of bortezomib on H2AK119ub levels in K562 and MOLM-13 cells. The cells were treated with 5 nM bortezomib (Bort) for 4 hours. Control-treated cells were incubated with DMSO. (A) Ponceau S-stained immunoblot of acid-extracted histones (left panel) and immunostained blot with anti-H2AK119ub antibody.

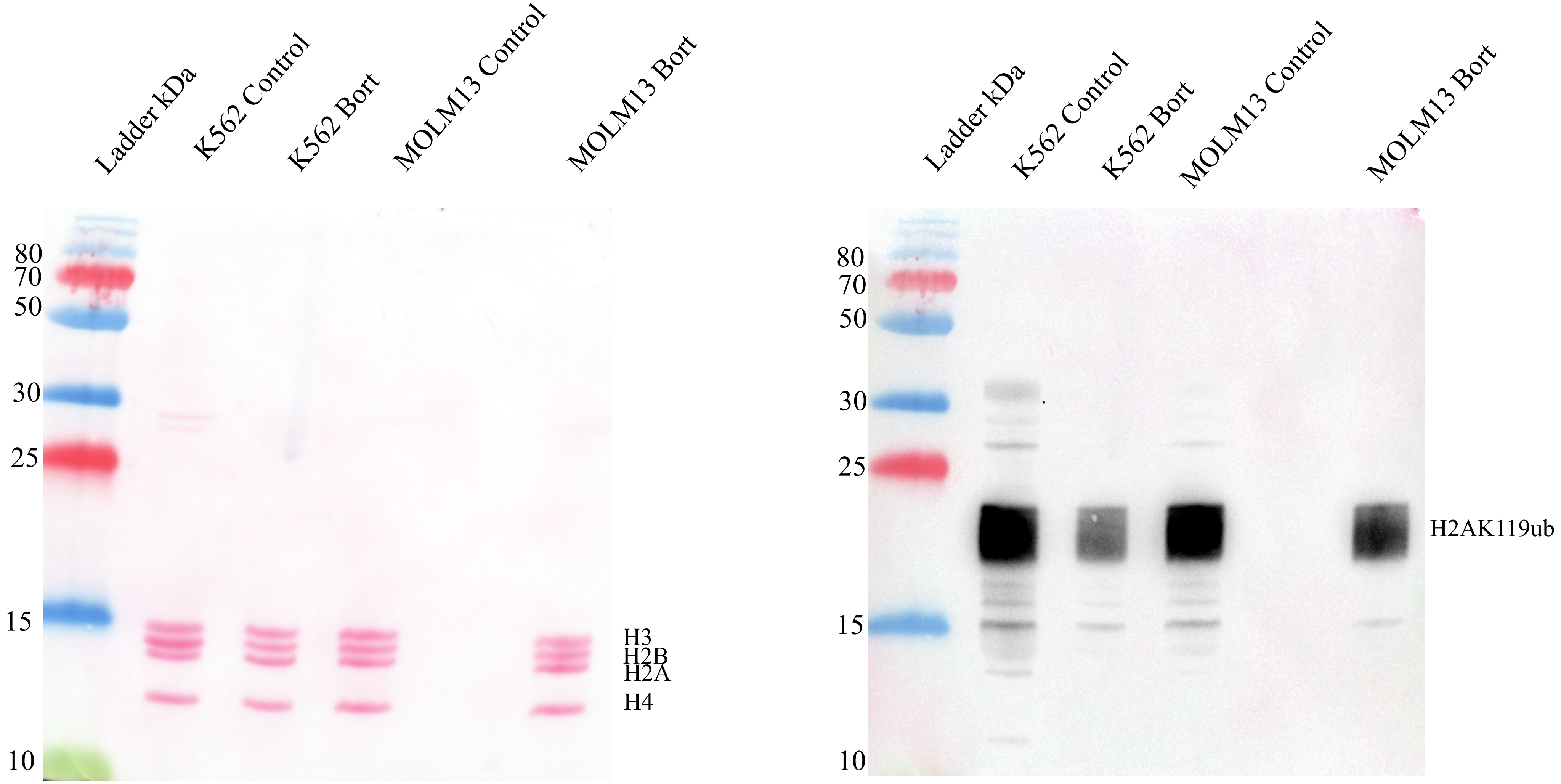

Biological Replicate 1

Figure S6B. Effect of bortezomib on H2AK119ub levels in K562 and MOLM-13 cells. The cells were treated with 5 nM bortezomib (Bort) for 4 hours. Control-treated cells were incubated with DMSO. (A) Ponceau S-stained immunoblot of acid-extracted histones (left panel) and immunostained blot with anti-H2AK119ub antibody.

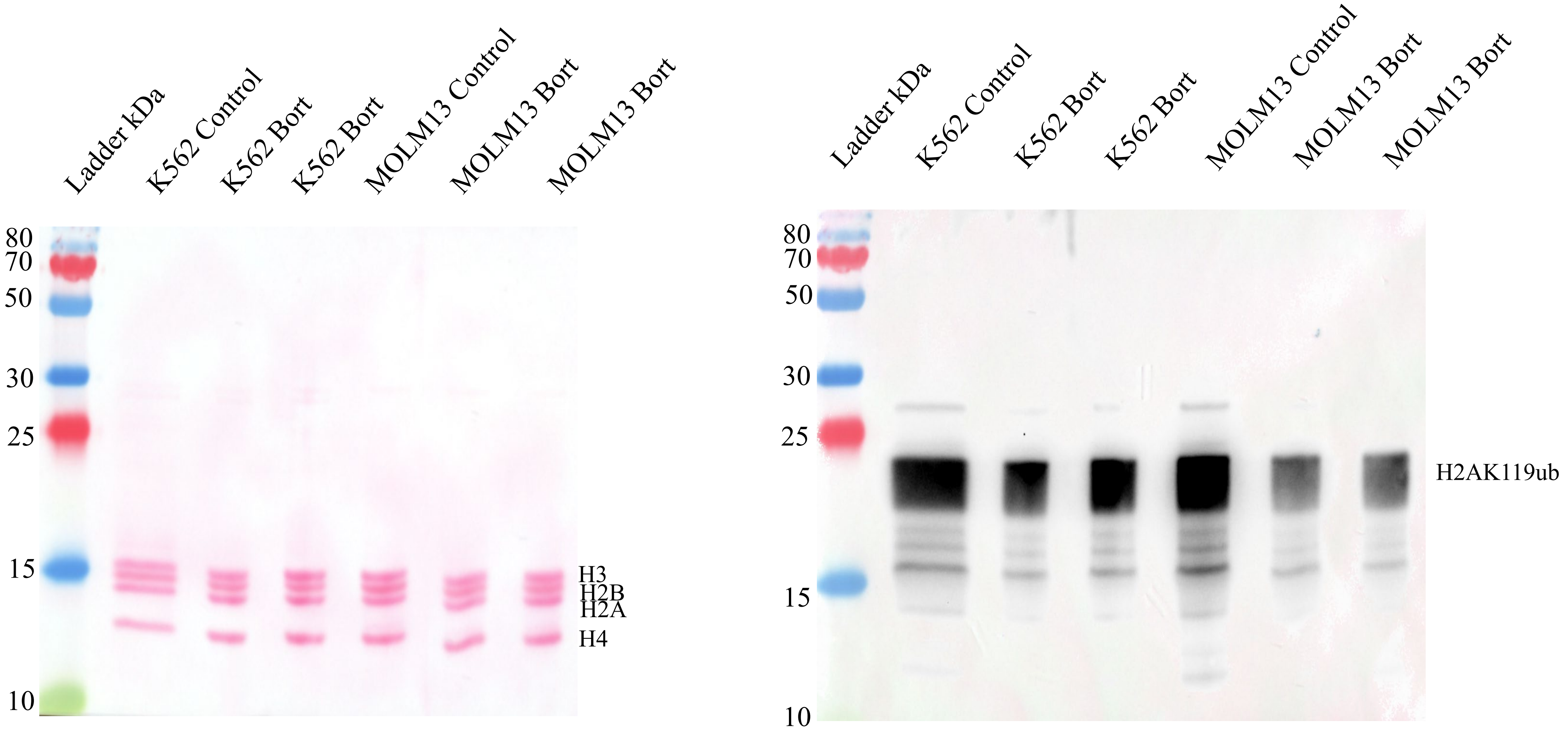

Biological Replicate 2

Figure S6C. Effect of bortezomib on H2AK119ub levels in K562 and MOLM-13 cells. The cells were treated with 5 nM bortezomib (Bort) for 4 hours. Control-treated cells were incubated with DMSO. (A) Ponceau S-stained immunoblot of acid-extracted histones (left panel) and immunostained blot with anti-H2AK119ub antibody.

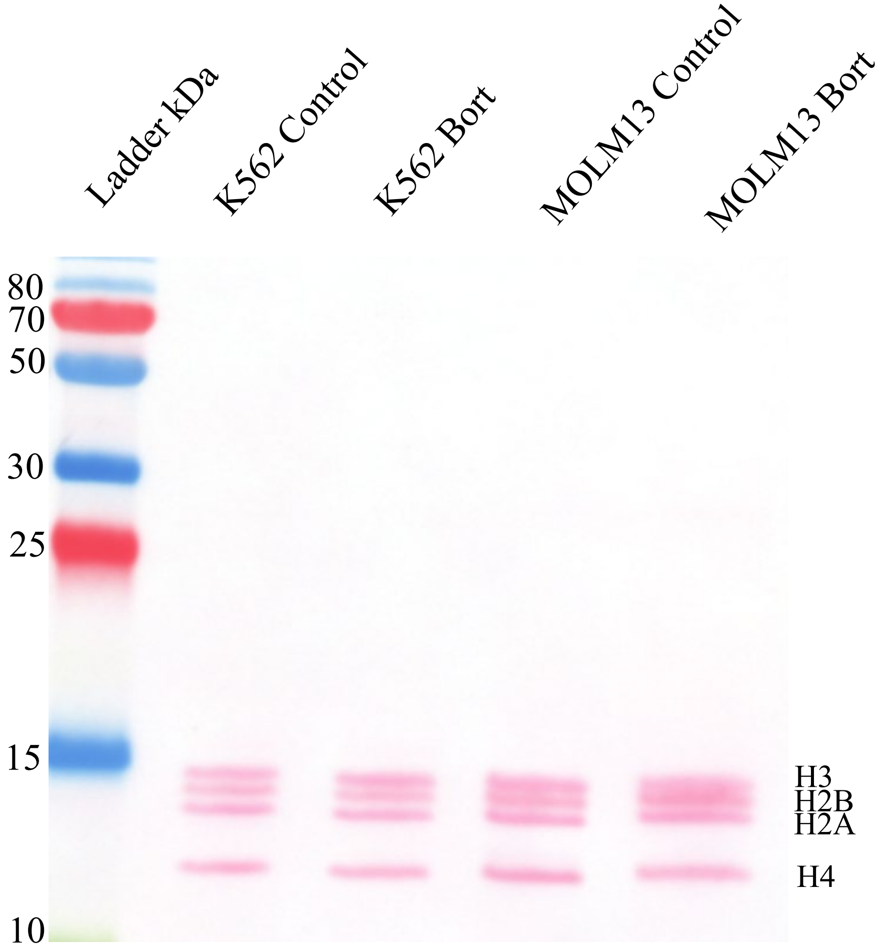

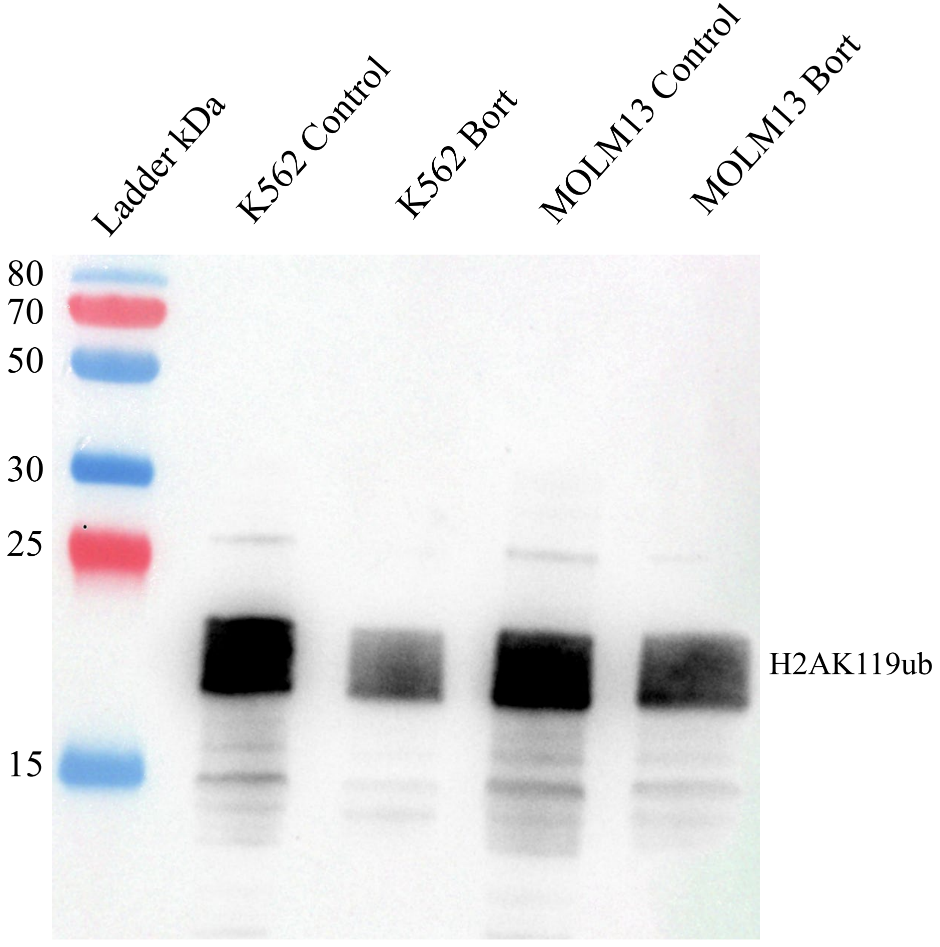

Biological Replicate 3

Figure S7. Effect of bortezomib on H2BK120ub levels in K562 cells. The cells were treated with 5 nM bortezomib for 2 and 4 hours. Control-treated cells were incubated with DMSO. Ponceau S-stained immunoblot of acid-extracted histones (left panel) and immunostained blot with anti-H2BK120ub antibody.

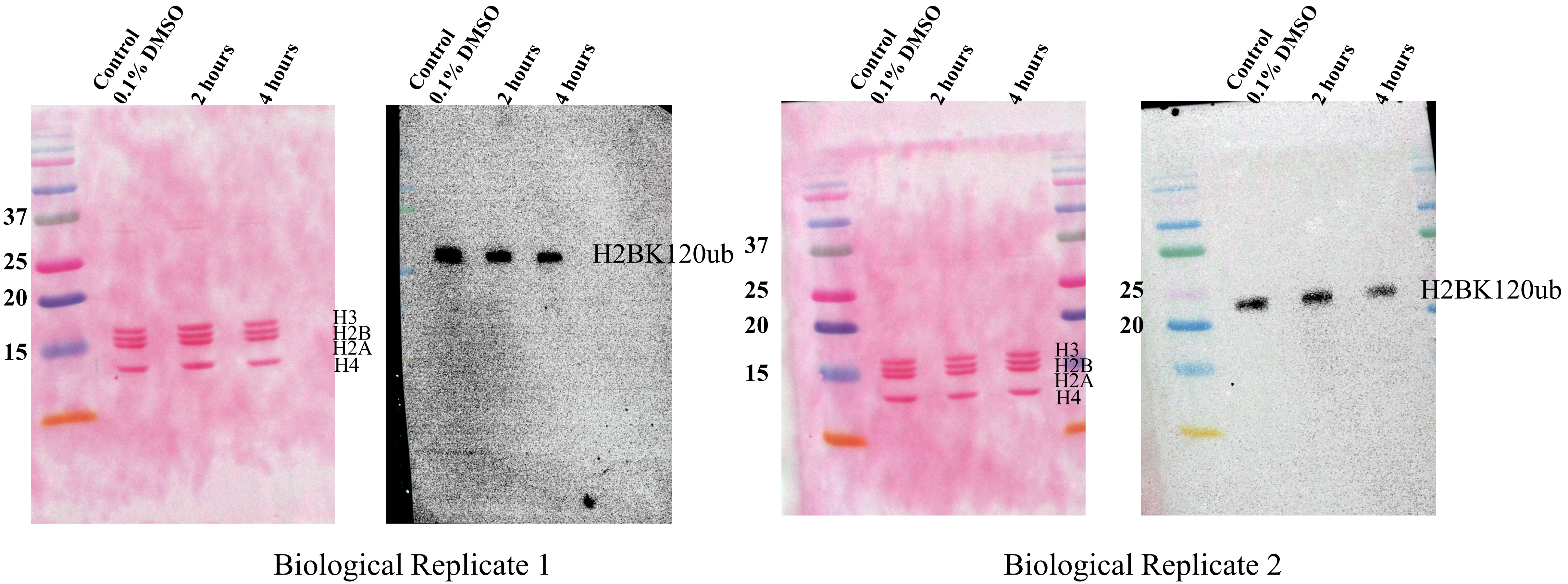

Figure S8A. Effect of bortezomib on H2BK120ub levels in MOLM-13 cells. The cells were treated with 5 nM bortezomib for 2 and 4 hours. Control-treated cells were incubated with DMSO. Ponceau S-stained immunoblot of acid-extracted histones (left panel) and immunostained blot with anti-H2BK120ub antibody. (Biological replicate 1)

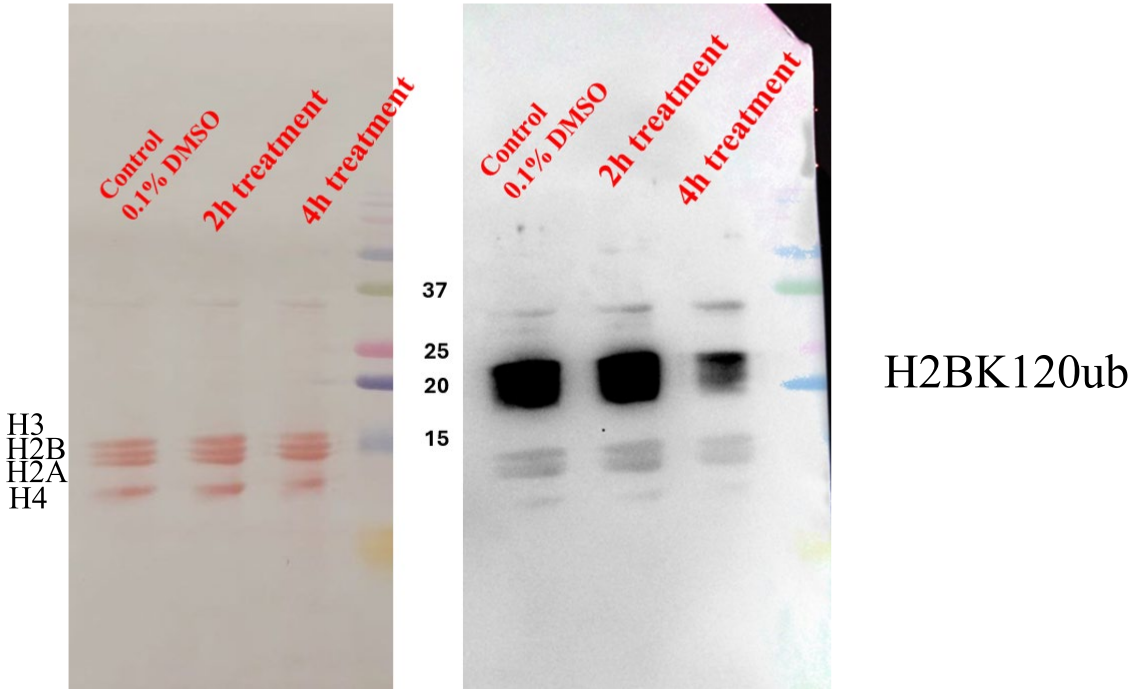

Figure S8B. Effect of bortezomib on H2BK120ub levels in MOLM-13 cells. The cells were treated with 5 nM bortezomib for 2 and 4 hours. Control-treated cells were incubated with DMSO. Ponceau S-stained immunoblot of acid-extracted histones (left panel) and immunostained blot with anti-H2BK120ub antibody. (Biological replicate 2 and 3)

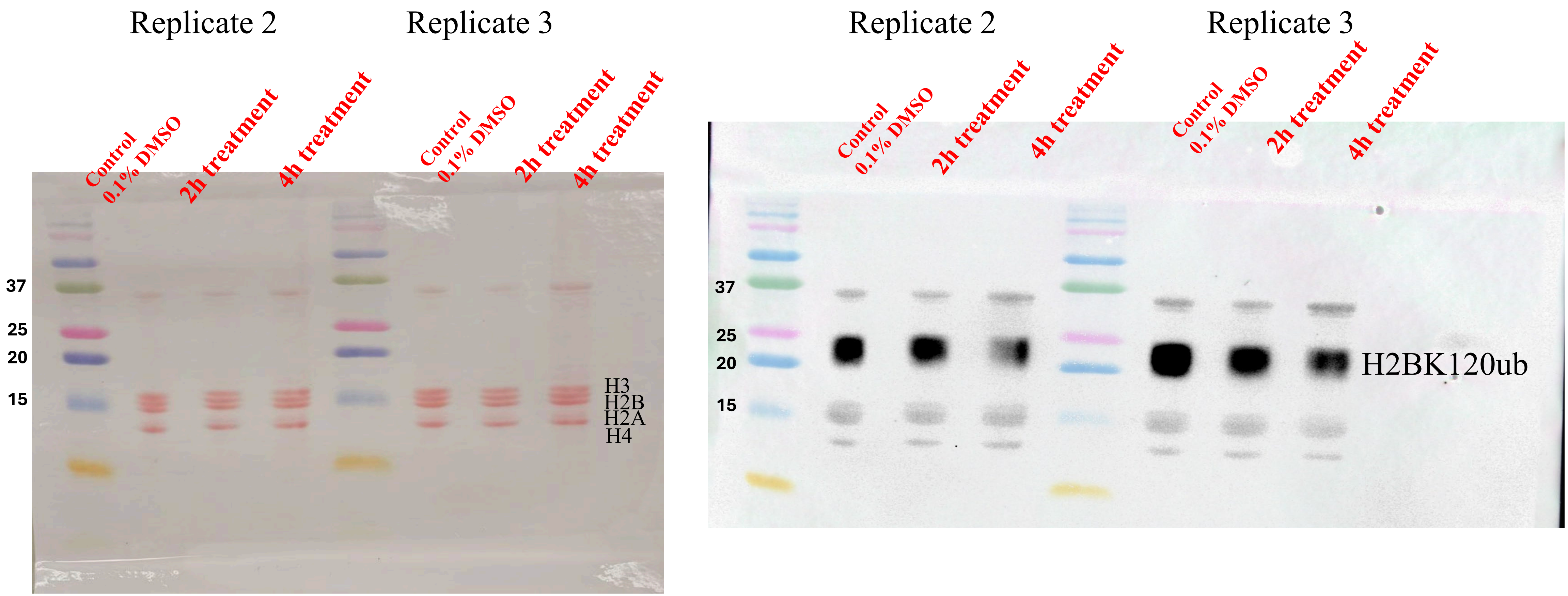

Figure S9. Effect of bortezomib on H3K79me2 levels in K562 cells. The cells were treated with 5 nM bortezomib for 2 and 4 hours. Control-treated cells were incubated with DMSO. Ponceau S-stained immunoblot of acid-extracted histones (left panel) and immunostained blot with anti-H3K79me2 antibody.

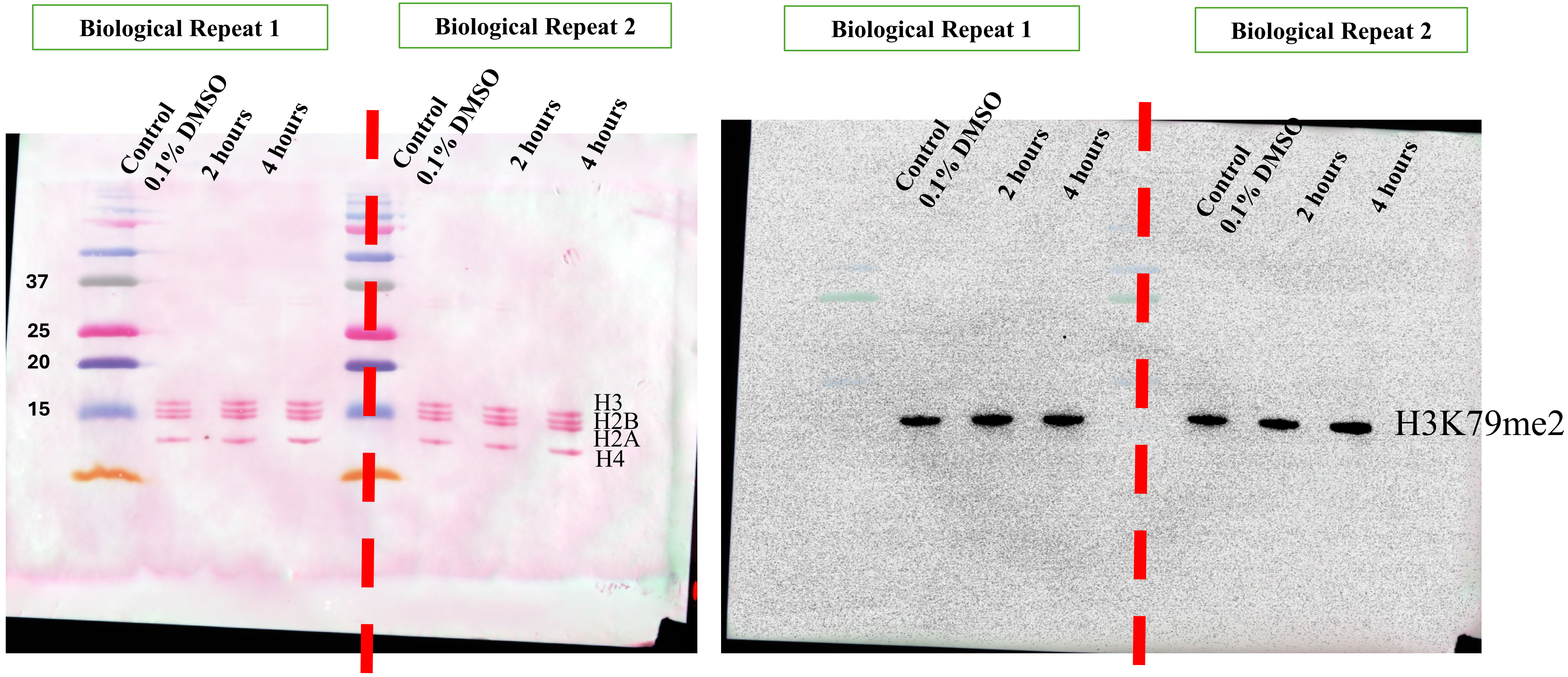

Figure S10A. Effect of bortezomib on H3K79me2 levels in MOLM-13 cells. The cells were treated with 5 nM bortezomib for 2 and 4 hours. Control-treated cells were incubated with DMSO. Ponceau S-stained immunoblot of acid-extracted histones (left panel) and immunostained blot with anti-H3K79me2 antibody.

Biological Replicate 1

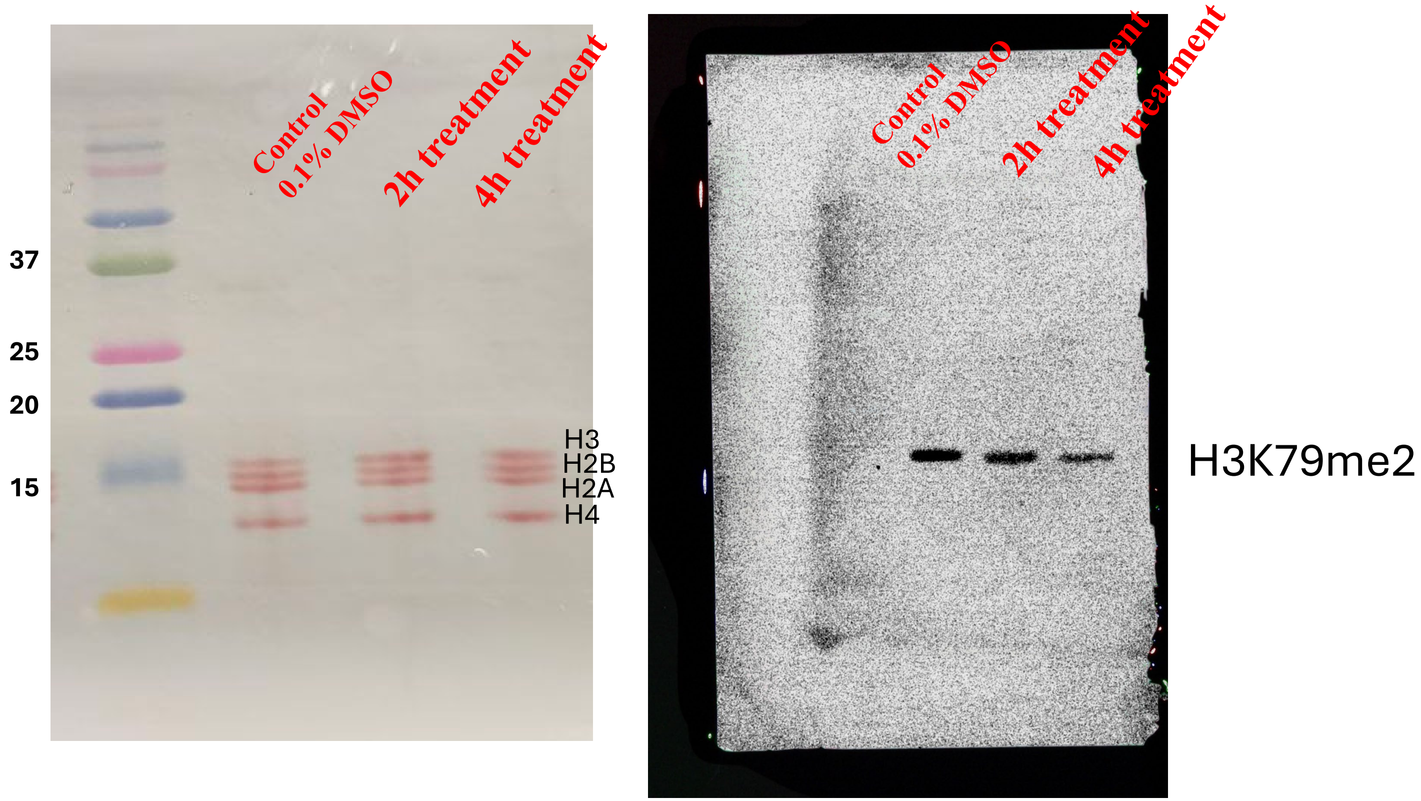

Figure S10B. Effect of bortezomib on H3K79me2 levels in MOLM-13 cells. The cells were treated with 5 nM bortezomib for 2 and 4 hours. Control-treated cells were incubated with DMSO. Ponceau S-stained immunoblot of acid-extracted histones (left panel) and immunostained blot with anti-H3K79me2 antibody.

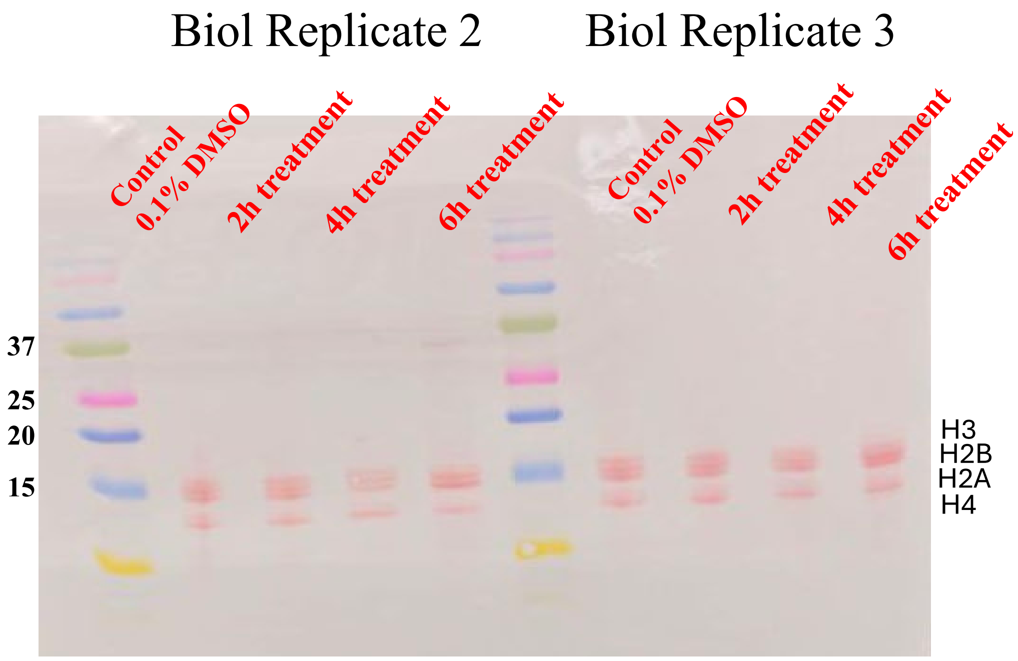

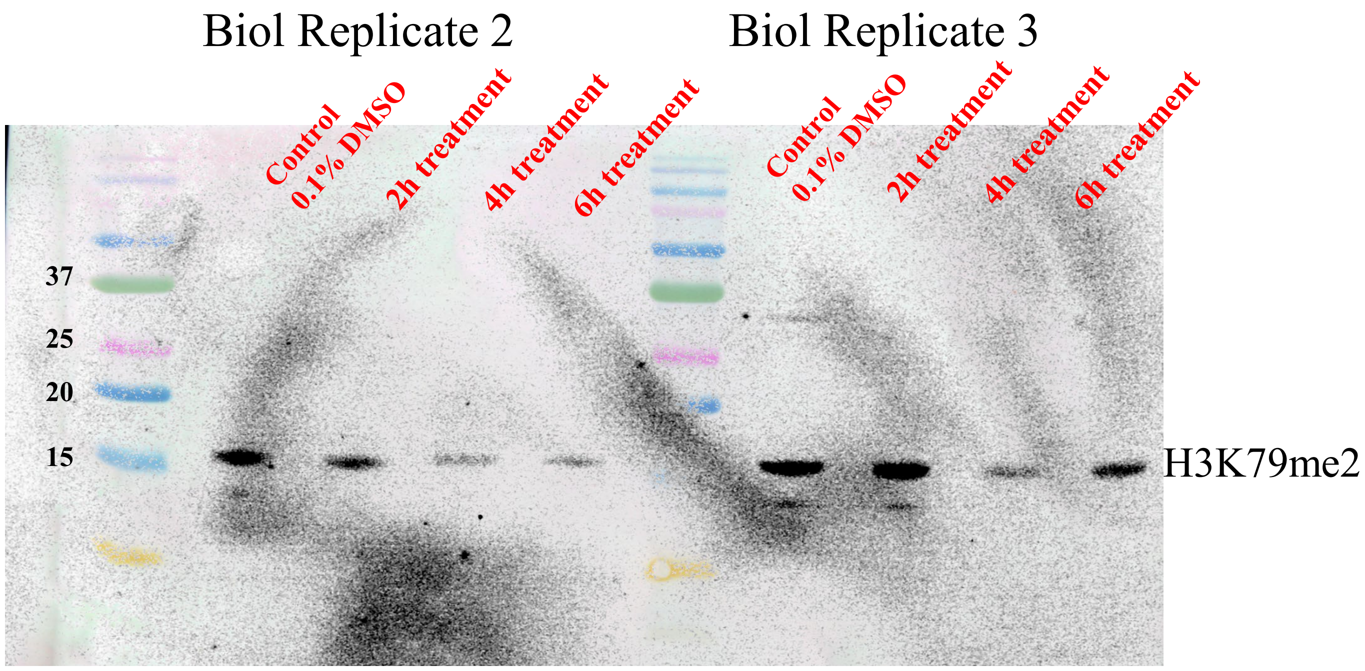

Figure S11A. Effect of bortezomib on H3K79me2 levels in MOLM-13 cells. The cells were treated with 5 nM bortezomib for 4 hours. Control-treated cells were incubated with DMSO. The Ponceau S-stained blot of histones resolved by AUT 15% PAGE (left panel), and the accompanying blot immunostained with antibodies against H3K79me2 (right panel) is shown. Biological replicate 1.

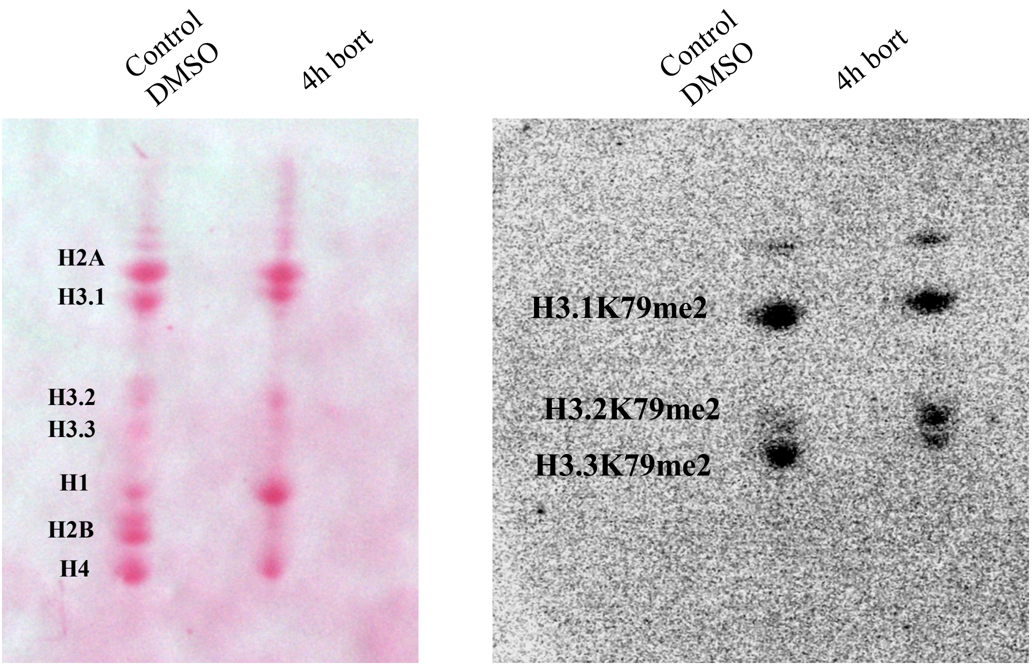

Figure S11B. Effect of bortezomib on H3K79me2 levels in MOLM-13 cells. The cells were treated with 5 nM bortezomib for 4 hours. Control-treated cells were incubated with DMSO. The Ponceau S-stained blot of histones resolved by AUT 15% PAGE (left panel), and the accompanying blot immunostained with antibodies against H3K79me2 (right panel) is shown.

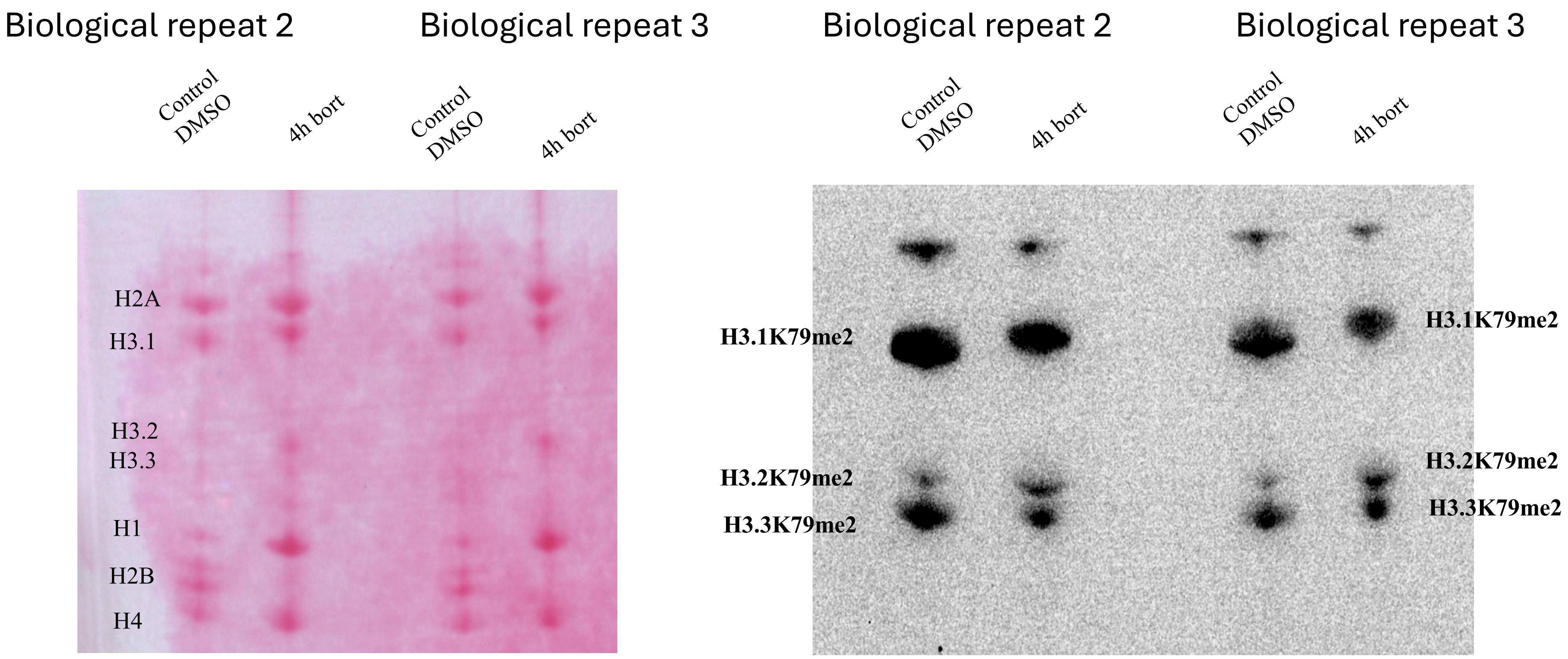

Figure S12. Effect of bortezomib on H3K36me3 levels in K562 cells. The cells were treated with 5 nM bortezomib for 2 and 4 hours. Control-treated cells were incubated with DMSO. Ponceau S-stained immunoblot of acid-extracted histones (left panel) and immunostained blot with anti-H3K36me3 antibody.

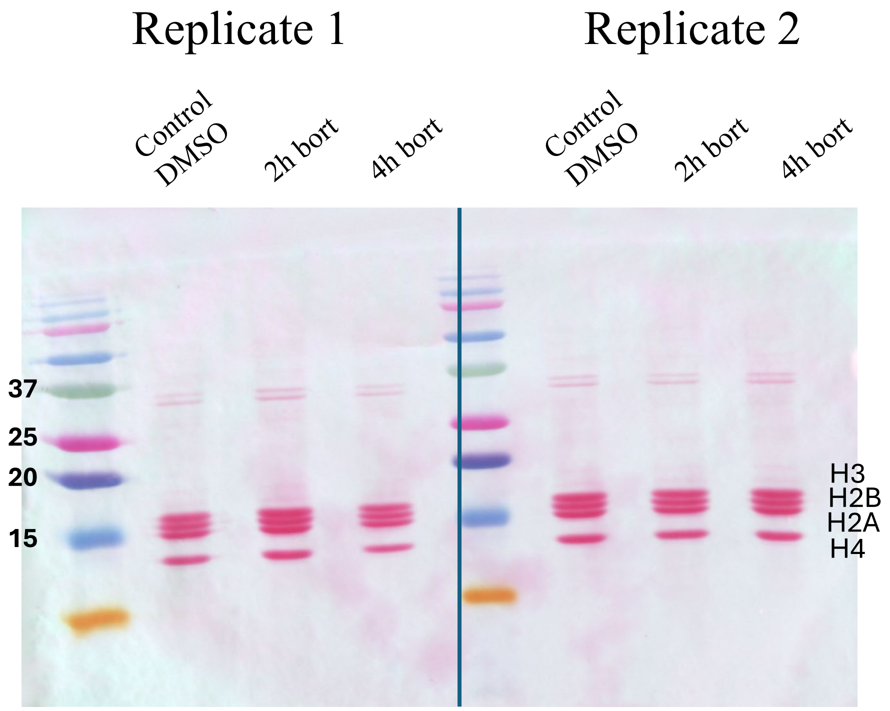

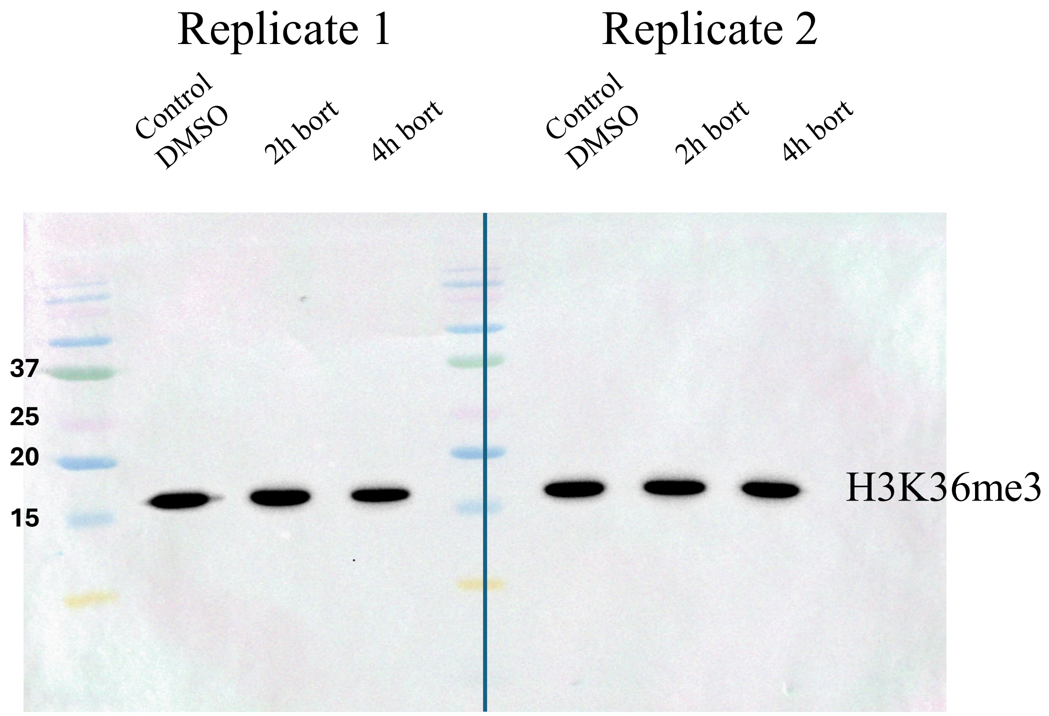

Figure S13A. Effect of bortezomib on H3K36me3 levels in MOLM-13 cells. The cells were treated with 5 nM bortezomib for 2 and 4 hours. Control-treated cells were incubated with DMSO. Ponceau S-stained immunoblot of acid-extracted histones (left panel) and immunostained blot with anti-H3K36me3 antibody. (biological replicate 1)

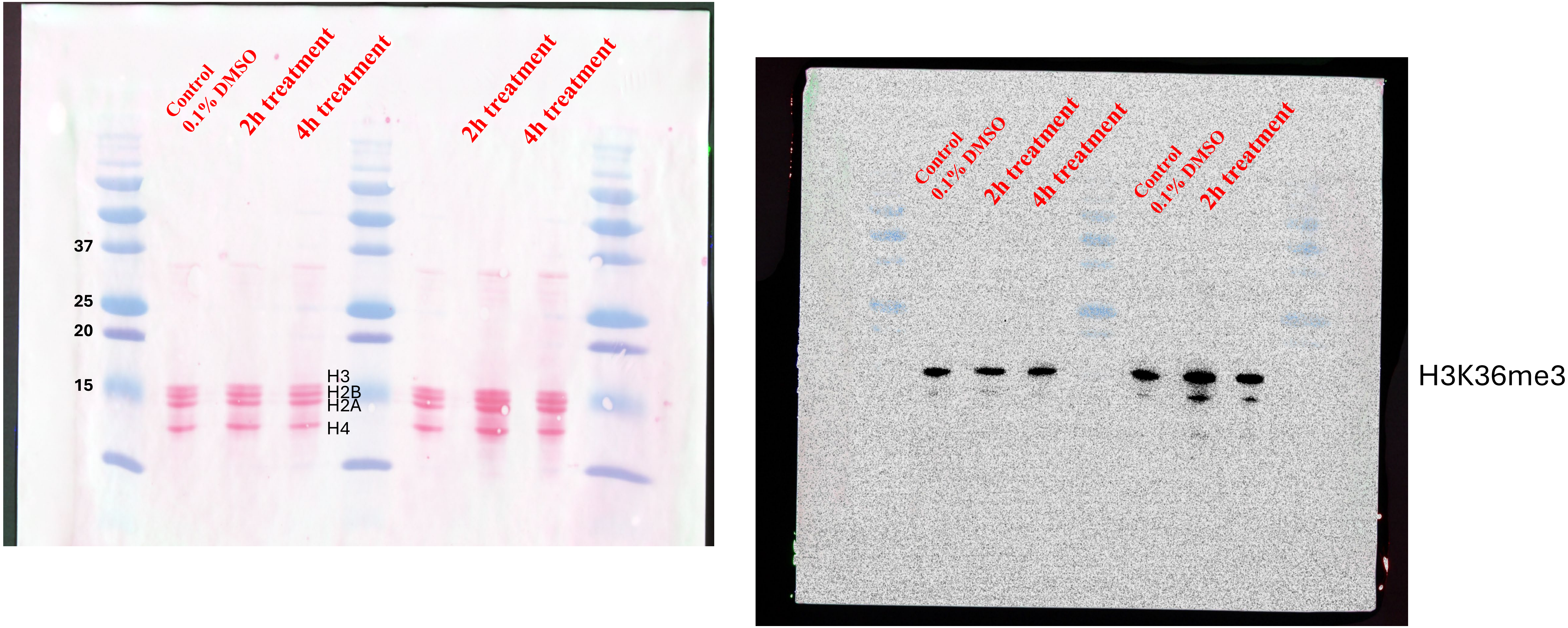

Figure S13B. Effect of bortezomib on H3K36me3 levels in MOLM-13 cells. The cells were treated with 5 nM bortezomib for 2 and 4 hours. Control-treated cells were incubated with DMSO. Ponceau S-stained immunoblot of acid-extracted histones (left panel) and immunostained blot with anti-H3K36me3 antibody. (Biological replicate 2 and 3)

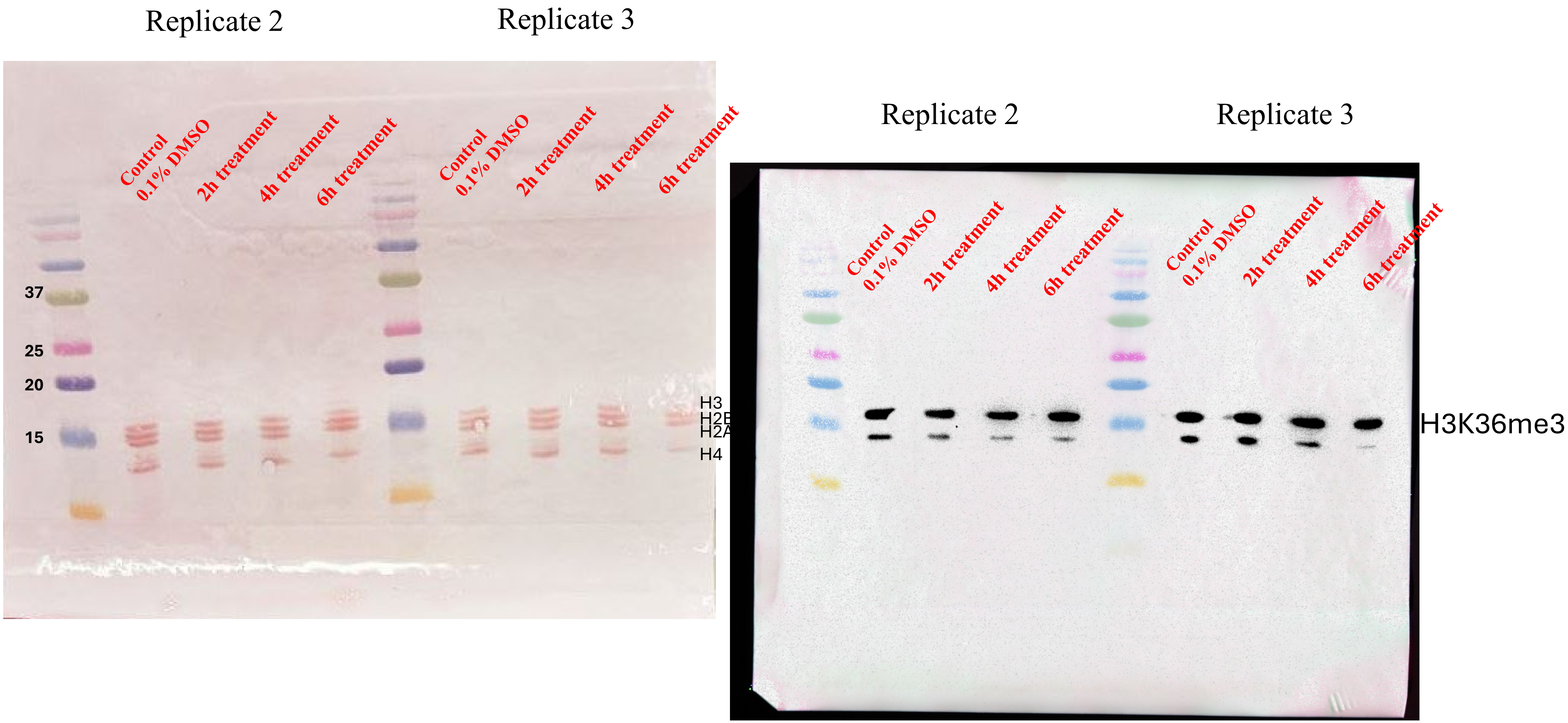

Figure S14A. Effect of ONX-0914 on H2BK120ub levels in MOLM-13 cells. The cells were treated with 50 nM ONX-0914 for 2 hours. Control-treated cells were incubated with DMSO. Ponceau S-stained immunoblot of acid-extracted histones (left panel) and immunostained blot with anti-H2BK120ub antibody. Biological replicate 1.

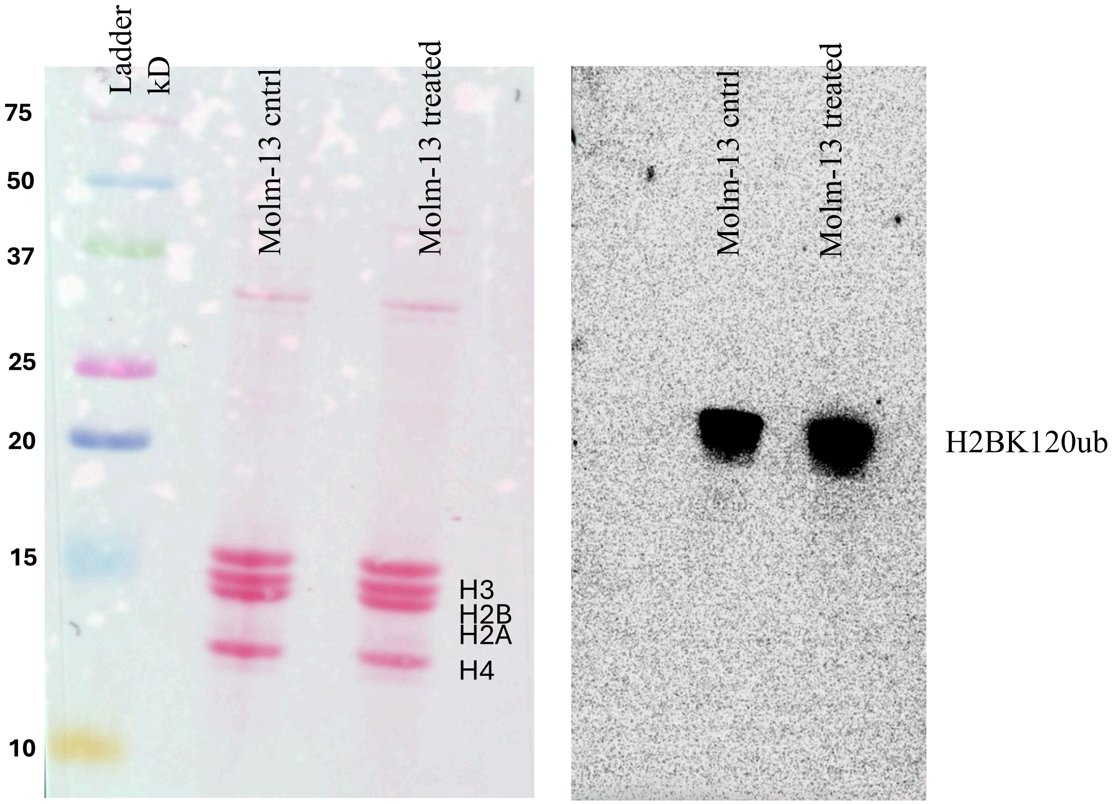

Figure S14B. Effect of ONX-0914 on H2BK120ub levels in K562 and MOLM-13 cells. The treatment/times shown are K562 control (200 nM/4 h), K562 ONX-0914-treated (200 nM/4 h), MOLM-13 control (200 nM/4 h), MOLM-13 ONX-0914-treated (200 nM/4 h), MOLM-13 control (50 nM/2 h), MOLM-13 ONX-0914-treated (50nM/2hr), MOLM-13 control (50 nM/4 h), and MOLM-13 ONX-0914-treated (50 nM/4 h). Control-treated cells were incubated with DMSO. Ponceau S-stained immunoblot of acid-extracted histones (left panel) and immunostained blot with anti-H2BK120ub antibody. Biological replicates 2 and 3 for MOLM-13 cells

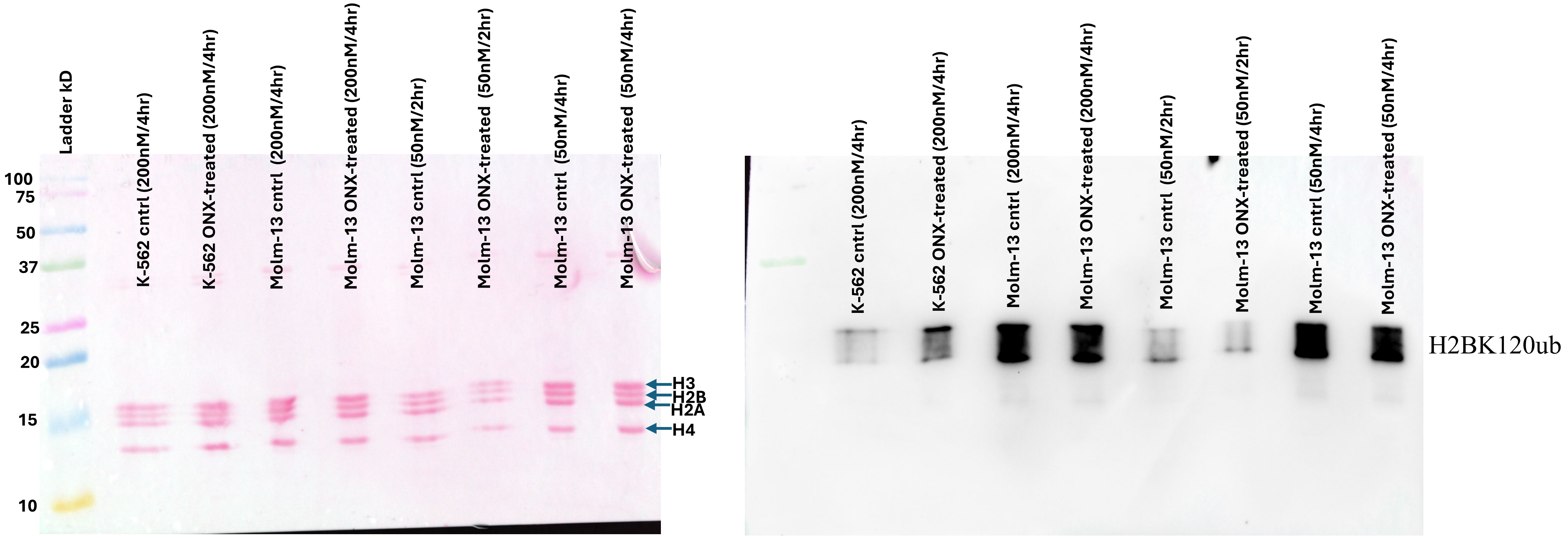

Figure S15. Effect of ONX-0914 on H2AK119ub levels in K562 and MOLM-13 cells. The treatment/times shown are K562 control (200 nM/4 h), K562 ONX-0914-treated (200 nM/4 h), MOLM-13 control (200 nM/4 h), MOLM-13 ONX-0914-treated (200 nM/4 h), MOLM-13 control (50 nM/2 h), MOLM-13 ONX-0914-treated (50nM/2hr), MOLM-13 control (50 nM/4 h), and MOLM-13 ONX-0914-treated (50 nM/4 h). Control-treated cells were incubated with DMSO. Ponceau S-stained immunoblot of acid-extracted histones (left panel) and immunostained blot with anti-H2AK119ub antibody.

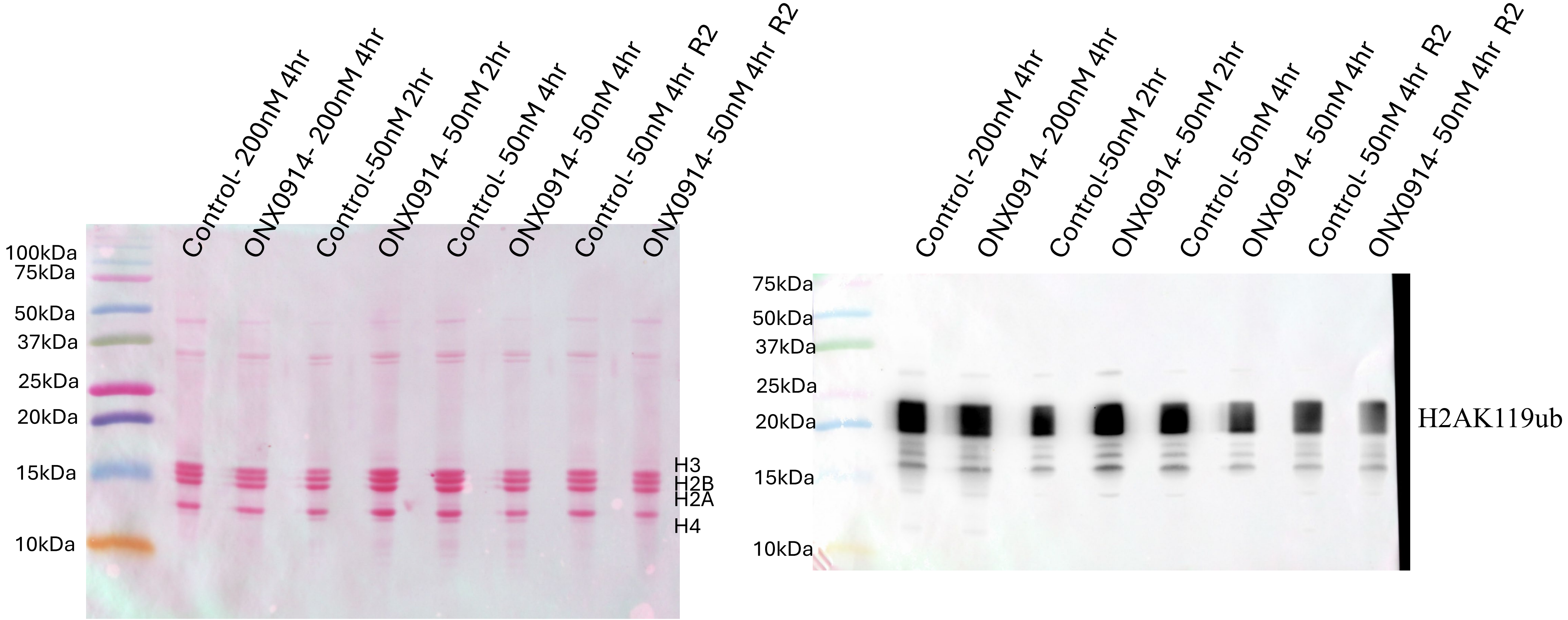

Figure S16A. Fractionation of bortezomib-treated and control K562 and MOLM-13 cells. The cells were treated with bortezomib (Bort) (50 nM for 2 hours). Control-treated cells were incubated with DMSO. Ponceau S-stained immunoblot of cell lysate and chromatin fraction is shown in the upper panel. The blots in the bottom panel were immunostained with anti-H2BK120ub antibodies.

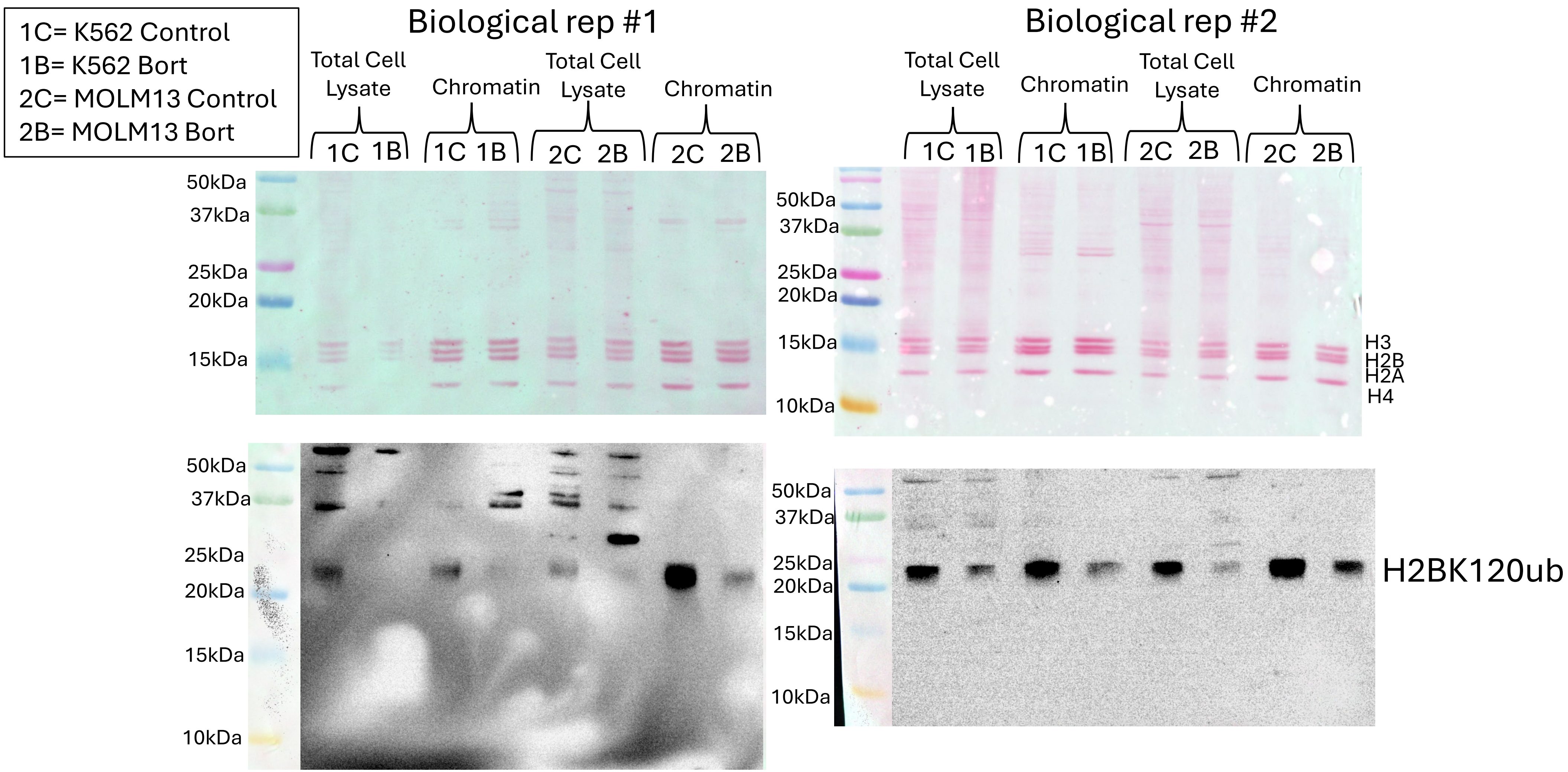

Figure S16B. Fractionation of bortezomib-treated and control K562 and MOLM-13 cells. The cells were treated with bortezomib (Bort) (50 nM for 2 hours). Control-treated cells were incubated with DMSO. Ponceau S-stained immunoblot of cell lysate and chromatin fraction is shown in the upper panel. The blots in the bottom panel were immunostained with anti-H2BK120ac antibodies.

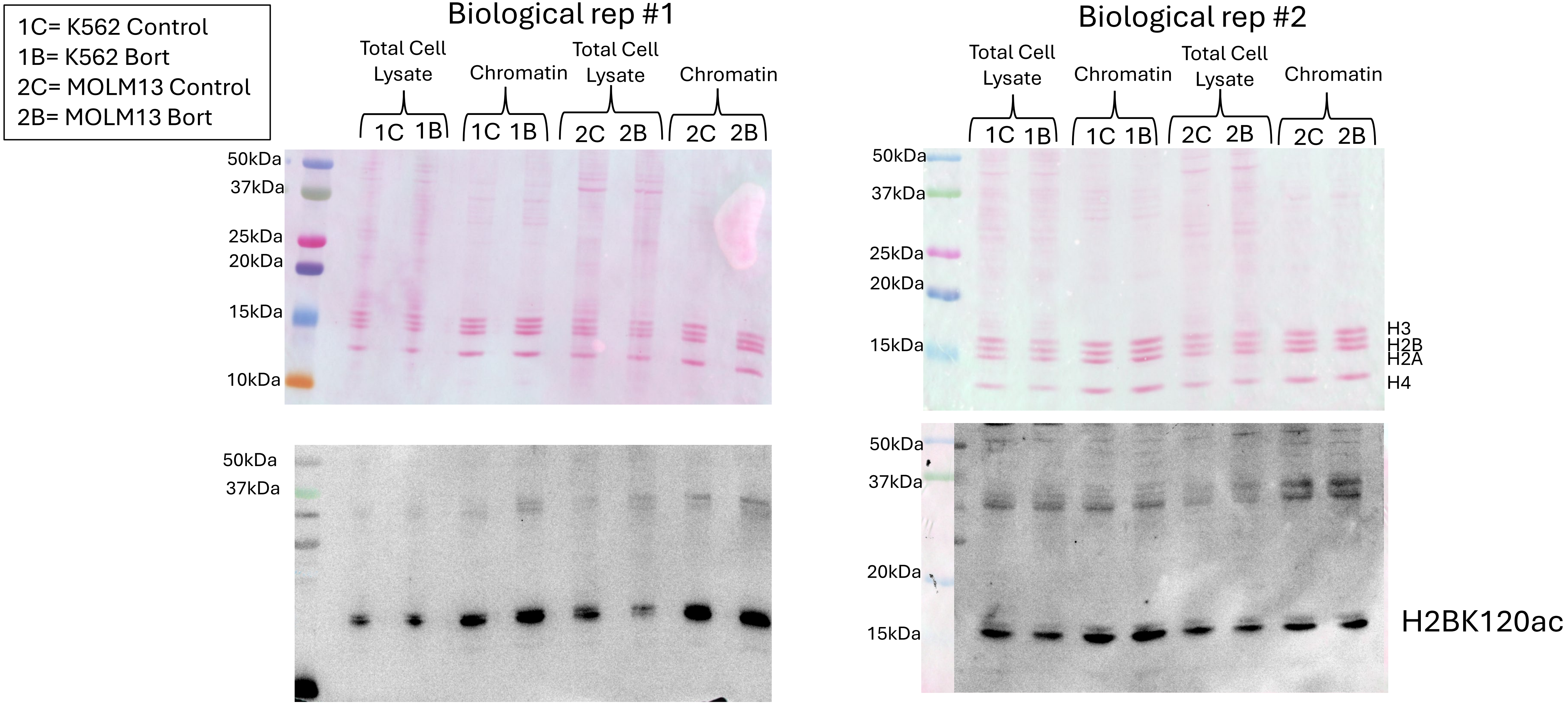

Table S1. Antibodies

Antibody Target	Company	Catalog number	Dilution	Host	Antibody Type
H3K79me2	Active motif	91399	1:1000	Rabbit	Recombinant
H3K36me3	Invitrogen	MA5-42755	1:1000	Rabbit	Recombinant
H3K4me3	Millipore	17-614	1:2000	Rabbit	Monoclonal
Ubiquitinated Histone H2B (Lys123)	Millipore Sigma	MABE1785	1:500	Mouse	Monoclonal
β-Actin	Millipore Sigma	A5316	1:10000	Mouse	Monoclonal
KMT4 (DOT1L)	Abcam	ab239358	1:1000	Rabbit	Monoclonal
Goat anti-Rabbit	Invitrogen	32460	1:2000	Goat	Polyclonal
Goat anti-Rabbit	Abcam	ab97051	1:50000	Goat	Polyclonal
Goat anti-Mouse	Invitrogen	31430	1:50000	Goat	Polyclonal
H2BK120ub1 antibody	Active Motif	39623	1:1000	Mouse	Monoclonal
Ubiquityl H2A (K119)	Cell Signalling	8240S	1:2000	Rabbit	Monoclonal
Beta actin	Invitrogen	A5441, AM4302	1:10000	Mouse	Monoclonal
H2B acetyl Lys 120	Active Motif	39119	1:2000	Rabbit	Polyclonal

Table S2. Sequence of primers

Primer target	Forward Sequence	Reverse Sequence
HOXA9- exon 2-3	CCCATCGATCCCAATAACCCA	AGTTCCAGGGTCTGGTGTTT
DOT1L - exon 19-20	GGAGAAGCTGTTGAAGGAGAAG	GCTCTTCTCTAGCTCCACAATG
MYC- exon 1-2	CAGTAGAAATACGGCTGCAC	TTCGGGTAGTGGAAAACCAG
MEIS1- exon 8-9	ACAAATATCATGAGGGCGTGG	GTGAGTCCCGTGTCTTGTG
PPIB- exon 1-2	CTCCGAACGCAACATGAAGG	CCAAAGATCACCCGGCCTAC
GAPDH- exon 8-9	CAACAGCGACACCCACTCCT	CACCCTGTTGCTGTAGCCAAA
